# Galectin-3 activates spinal microglia to induce inflammatory nociception in wild type but not in mice modelling Alzheimer’s disease

**DOI:** 10.1038/s41467-023-39077-1

**Published:** 2023-06-22

**Authors:** George Sideris-Lampretsas, Silvia Oggero, Lynda Zeboudj, Rita Silva, Archana Bajpai, Gopuraja Dharmalingam, David A. Collier, Marzia Malcangio

**Affiliations:** 1 Wolfson Centre for Age-Related Diseases, King’s College London, London, United Kingdom; 2 Eli Lilly & Company, Surrey, 8 Arlington Square West, Bracknell, RG12 1PU United Kingdom

**Keywords:** Neuroimmunology, Alzheimer's disease, Chronic pain, Microglia

## Abstract

Musculoskeletal chronic pain is prevalent in individuals with Alzheimer’s disease (AD); however, it remains largely untreated in these patients, raising the possibility that pain mechanisms are perturbed. Here, we utilise the TASTPM transgenic mouse model of AD with the K/BxN serum transfer model of inflammatory arthritis. We show that in male and female WT mice, inflammatory allodynia is associated with a distinct spinal cord microglial response characterised by TLR4-driven transcriptional profile and upregulation of P2Y12. Dorsal horn nociceptive afferent terminals release the TLR4 ligand galectin-3 (Gal-3), and intrathecal injection of a Gal-3 inhibitor attenuates allodynia. In contrast, TASTPM mice show reduced inflammatory allodynia, which is not affected by the Gal-3 inhibitor and correlates with the emergence of a P2Y12^−^ TLR4^−^ microglia subset in the dorsal horn. We suggest that sensory neuron-derived Gal-3 promotes allodynia through the TLR4-regulated release of pro-nociceptive mediators by microglia, a process that is defective in TASTPM due to the absence of TLR4 in a microglia subset.

## Abstract (web summary)

In inflammatory arthritis, pain neurons communicate with spinal cord microglia to establish nociception. Here, the authors show that this communication is mediated by pain neurons releasing galectin-3, which activates microglia through TLR4. In a mouse model of Alzheimer’s disease, pain is attenuated because microglia lack expression of TLR4.

## Introduction

Musculoskeletal disorders are characterised by moderate to severe chronic pain, which is the result of tissue or nerve damage and defined as nociceptive or neuropathic pain. Furthermore, a recent classification of musculoskeletal pain has introduced nociplastic pain to include pain in tissues without pathology that can be perceived as painful, highlighting its dynamic relationship with mood and cognition^1,2^. Indeed, musculoskeletal disorders, where nociplastic pain is typically present, are often presented by patients with cognitive disabilities^3^. Of note, musculoskeletal painful conditions are the second most prevalent comorbidity in individuals with Alzheimer’s disease (AD)^4^. Untreated chronic pain contributes to psychiatric symptoms of dementia^5^, while AD is considered a risk factor for the under-treatment of pain^6^. Therefore, a better understanding of the interplay between musculoskeletal chronic pain and AD might lead to improved mechanism-based analgesic treatments.

Rheumatoid arthritis (RA) is a chronic musculoskeletal inflammatory disease characterised by synovial inflammation, and mid-life RA increases the risk of developing AD^7^. Despite optimal control of the joint inflammation, people with RA identify pain as the most debilitating symptom^8^. Specifically, inflammatory arthritis pain is initially driven by synovial inflammation and sensitisation of sensory neurons innervating the joint (peripheral sensitisation) and then maintained by concomitant neuronal plasticity at the first sensory synapse within the spinal cord (central sensitisation). Preclinical models of RA are instrumental in determining the mechanisms behind nociceptive processing in inflammatory arthritis. In particular, in models of arthritis, spinal cord microgliosis and increased cytokine levels, in response to inflammation-evoked sensory neuron excitation, are closely linked to nociception^9,10^. Furthermore, in the K/BxN serum transfer (ST) models of inflammatory arthritis, neuro-immune interactions are integral to nociceptive signalling at the dorsal root ganglia and spinal cord dorsal horn^11–13^. Mice are passively immunised with serum containing anti-glucose-6-phosphate isomerase (anti-G6PI) antibodies that induce autoimmune inflammatory attack exclusively in the distal joints where anti-G6PI antibodies bind to G6PI expressed on the cartilage surface and mediate activation of the innate immune system^14^. The insurgence of joint inflammation after K/BxN ST induces significant hind paw mechanical hypersensitivity that persists after the resolution of joint swelling^11,13^.

To study possible dysregulation of pain mechanisms in AD, we combined the K/BxN ST model with the TASTPM transgenic mouse model of AD since 6-month-old TASTPM mice display both sensory and cognitive deficits^15^. At the tissue level, TASTPM brains exhibit AD neuropathology, including amyloid deposition associated with microgliosis and reactive astrocytes^15^. In the spinal cord, microgliosis is prevalent in 6-month-old mice, preceding the formation of plaques which results from progressive amyloid build-up in 12-month-old TASTPM^15^. Despite the AD-like pathology, blood–brain barrier (BBB) integrity appears normal in TASTPM mice as brain penetration of permeability markers such as sodium fluorescein is not enhanced in TASTPM^16^.

In this study, by comparing the development of K/BxN ST inflammatory arthritis in wild-type (WT) and TASTPM mice, we investigate microglia responses in inflammatory arthritis and assess whether amyloid-β pathology results in the alteration of neuro-immune communication in inflammatory pain mechanisms. We show that inflammatory allodynia and dorsal horn microglial activation are attenuated in male and female TASTPM mice compared to WT. Inflammatory allodynia is associated with a TLR4-driven transcriptional profile and upregulation of P2Y12 in WT microglia, whilst in TASTPM, a subset of microglia does not express TLR4. In addition, both WT and TASTPM microglia upregulate the TLR4 ligand galectin-3 (Gal-3), which is also expressed and released by nociceptive fibres in the dorsal horn. In WT, but not TASTPM mice, inflammatory allodynia is reversed by intrathecal delivery of a Gal-3 inhibitor, and Gal-3 is pro-nociceptive in WT but not TLR4 KO mice. Our data indicate that a Gal-3 and TLR4 interaction mediates neuro-microglia communication in inflammatory allodynia, and such a pro-nociceptive pathway is flawed in TASTPM mice.

## Results

### Attenuation of inflammatory arthritis allodynia in TASTPM mice

**Fig. 1:**
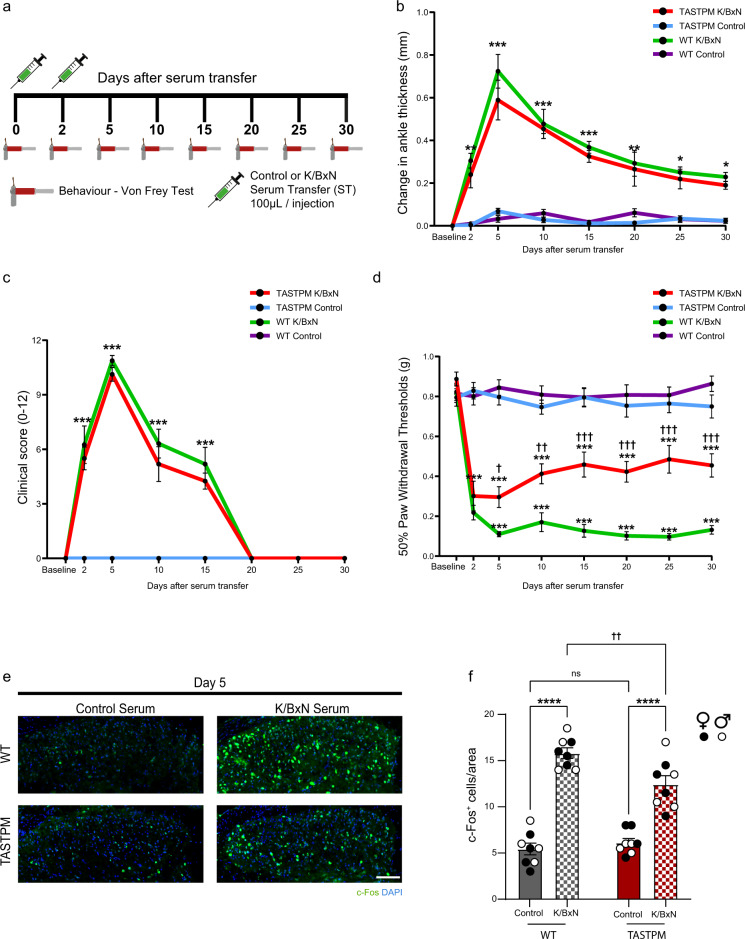
TASTPM mice develop less allodynia than WT in the K/BxN serum transfer model of arthritis. **a** Schematic overview of study design. **b** Ankle thickness and **c** clinical scores of fore and hind paws in WT and TASTPM. **d** Reduced mechanical hypersensitivity (PWT paw withdrawal thresholds) in TASTPM after K/BxN ST. Data represent mean ± SEM, *n* = 12 mice per group. *p < 0.05, ***p < 0.001 compared to same-day WT or TASTPM Control ST, †*p* < 0.05, ††*p* < 0.01, †††*p* < 0.001 compared to same-day WT K/BxN ST, two-way RM ANOVA, Tukey’s multiple comparisons test. **e** Representative images of c-Fos^+^ neurons in dorsal horns. Scale bar, 100 μm. **f** Quantification of c-Fos^+^ neurons. Data represent mean ± SEM, *n* = 8 mice per group (4 per sex). ns not significant, ****p < 0.0001, ††*p* = 0.0073. Two-way ANOVA, Tukey’s multiple comparisons test.

In both WT and TASTPM male and female mice, passive immunisation with K/BxN serum transfer (ST) but not control ST (Fig. 1a), resulted in development of ankle joint swelling and clinical signs of arthritis (clinical score) from day 2 after K/BxN ST, which peaked at day 5 and slowly subsided thereafter, approaching recovery by day 20 onwards (Fig. 1b, c and Supplementary Fig. 1a). Consistent with our previous report^11,17^, male and female WT mice displayed significant hind paw mechanical hypersensitivity at day 2 K/BxN ST, which persisted beyond attenuation of joint swelling and remained significant up to day 30 K/BxN ST (Fig. 1d and Supplementary Fig. 1b). However, in male and female TASTPM mice, we observed that whilst joint swelling and clinical score developed and resolved like in WT (Fig. 1b, c and Supplementary Fig. 1c), mechanical hypersensitivity was less severe than in WT at day 5 K/BxN ST and remained weaker for up to day 30 K/BxN ST (Fig. 1d and Supplementary Fig. 1d). Thus, K/BxN-induced allodynia was sex-dependent in neither WT nor TASTPM and we reasoned those potential peripheral changes in the joint would unlikely account for milder inflammatory allodynia in TASTPM and considered central mechanisms instead. We focussed on the first sensory synapse in the dorsal horn of the spinal cord and began with quantification of c-Fos, a marker of neuronal activity following noxious stimulation, at two points after K/BxN ST: day 5, at peak allodynia in WT but less severe allodynia in TASTPM, and day 30, at persistent allodynia in WT but significant recovery of allodynia in TASTPM. We observed that in both WT and TASTPM superficial laminae, c-Fos expression was significantly higher at day 5 but not at day 30 K/BxN ST (Fig. 1e, f and Supplementary Fig. 1e, f). It is plausible that at day 30 K/BxN ST, primary afferent activation of dorsal horn neurons continues but may no longer be identified using c-Fos as a marker. However, consistent with different extent of allodynia at day 5 K/BxN ST, c-Fos expression was lower in TASTPM than WT K/BxN ST (Fig. 1f). Considering that first sensory synapse activity can trigger the release of inflammatory mediators and phagocytosis in microglia^18^, we next examined dorsal horn (DH) microglial response at day 5 and 30 K/BxN ST.

### Dorsal horn microglia activation state correlates with K/BxN ST allodynia in WT and TASTPM

**Fig. 2:**
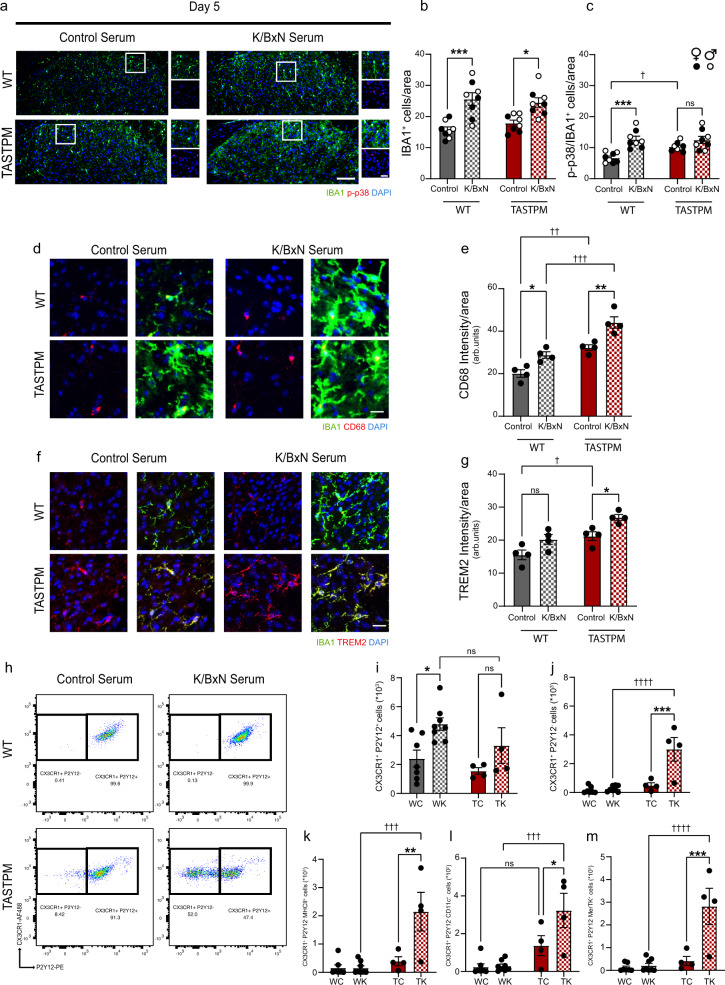
Dorsal horn microglia express a distinct set of markers in TASTPM at Day 5 K/BxN ST. **a** Representative images of p-p38^+^ IBA1^+^ cells in dorsal horns (DH). Scale bar, 100 μm. **b**, **c** Quantification of IBA1^+^ and p-p38^+^ IBA1^+^ microglia. Data represent mean ± SEM, *n* = 8 mice per group (4 per sex). **d** Representative images of CD68^+^ IBA1^+^ cells in DH. Scale bar, 20 μm. **e** Quantification of CD68 intensity in microglia in DH. Scale bar, 10 μm. **f** Representative images of TREM2/IBA1^+^ cells in DH. Scale bar, 10 μm. **g** Quantification of TREM2 intensity in microglia in DH. **h** Representative scatterplots of microglia sorted from DH. Cells were gated on CD45^low^, CD11b^+^, CX3CR1^+^. Data represent mean ± SEM, *n* = 4 mice per group. **i**, **j** Quantification of P2Y12^+^ and P2Y12^−^ microglia in DH. **k**–**m** Quantification of MHCII^+^, CD11c^+^, and MerTK^+^, in P2Y12^−^ microglia in DH. Data represent mean ± SEM, *n* = 7 (WT Control ST), *n* = 8 (WT K/BxN ST), and *n* = 4 (TASTPM Control and K/BxN ST). WC = wild type control serum; WK = wild type K/BxN serum; TC = TASTPM control serum; TK = TASTPM K/BxN serum. ns not significant, *p < 0.05, **p < 0.01, ***p < 0.001, ****p < 0.0001, †*p* < 0.05, ††*p* < 0.01, †††*p* < 0.001, ††††*p* < 0.0001. Two-way ANOVA, Tukey’s multiple comparisons test.

Microglia are endowed with the ability to sense and regulate microenvironments as local cues can shape their responses^19^. Hence, to examine whether K/BxN ST allodynia was associated with DH microglial response to neuronal activity, we quantified co-expression of IBA1 and p-p38 (phosphorylated p38), a marker of microglial response to noxious stimulation^20,21^. In male and female WT, total microglia (IBA1^+^ cells) and activated microglia (IBA1/p-p38^+^ cells) numbers were higher in K/BxN ST compared to control ST dorsal horns at both day 5 and day 30 (Fig. 2a–c and Supplementary Fig. 2a–c). However, in male and female TASTPM dorsal horn, K/BxN-associated changes in microglia were detectable at day 5 but not at day 30 K/BxN ST compared to control ST (Fig. 2a, b and Supplementary Fig. 2a, b). Furthermore, p-p38 expression was higher in microglia in TASTPM compared to WT control ST (Fig. 2a, c) but did not increase at either day 5 or day 30 K/BxN ST (Fig. 2c and Supplementary Fig. 2c). These results indicate a correlation between K/BxN ST allodynia and DH microglial response in a sex-independent manner. First, in WT, day 5 allodynia was associated with neuronal and microglial activation and in TASTPM, less severe day 5 allodynia was associated with a milder response of microglia. Second, at day 30, allodynia, which persisted in WT and recovered in TASTPM, was associated with microglial changes in WT but not TASTPM.

Since microglia are promptly involved in dorsal horn mechanisms underlying the development of allodynia after peripheral damage^22^, we selected day 5 K/BxN ST as the time point for further characterisation in WT and TASTPM. Thus, we examined the expression of CD68, a lysosomal marker for phagocytic microglia, which is upregulated in response to insoluble protein aggregate formations. We observed that CD68 expression was higher in TASTPM than WT control ST and increased above control levels in both WT and TASTPM K/BxN ST (Fig. 2d, e). These data suggest that both WT and TASTPM microglia were phagocytic at day 5 after K/BxN ST and TASTPM microglia were already involved in phagocytosis under control conditions. Since in TASTPM spinal cord plaques become visible at 12 months of age, it is plausible that 6-month-old TASTPM microglia upregulate CD68 in response to a rise of soluble amyloid-β in a similar fashion to hippocampal microglia^23^. We deem less plausible that K/BxN serum acts directly on spinal cord microglia since the BBB is intact in TASTPM mice and IgG does not readily cross the blood–spinal cord barrier^16,24^. Furthermore, AD brain microglia are known to downregulate P2Y12^25^ and upregulate TREM2^26^ and CD11c, which appears in the disturbance of homoeostasis^27^ and MerTK, which is observed in the vicinity of amyloid plaques^28^. Therefore, we examined the expression of these microglial markers in WT and TASTPM K/BxN ST. We observed that in WT, immunostaining of TREM2 in IBA1^+^ microglia was comparable in K/BxN ST and control ST (Fig. 2f, g), whereas TASTPM microglia expressed higher levels of TREM2 than WT under control conditions and TREM2 expression was further increased in K/BxN ST conditions (Fig. 2f, g). Then, using flow cytometry to examine the expression of P2Y12, CD11c, MHCII and MerTK in microglia (CD45^low^ CD11b^+^ CX3CR1^+^ population, strategy reported in Supplementary Fig. 3), we observed that WT CX3CR1^+^ cells showed distinct expression patterns from TASTPM. Specifically, in WT, but not TASTPM, CX3CR1^+^ P2Y12^+^ cell numbers were higher in K/BxN ST compared to control ST (Fig. 2h, i). Strikingly, TASTPM but not WT microglia, were heterogenous as we identified a subset of microglia defined by no P2Y12 expression (CD45^low^ CD11b^+^ CX3CR1^+^ P2Y12^−^ cells) (Fig. 2j), which co-expressed MHCII, CD11c and MerTK (Fig. 2k–m). This population of parenchymal CD11c^+^ microglia was detected mostly in TASTPM K/BxN ST, whereas few microglia showed CD11c expression in WT and TASTPM Control ST and WT K/BxN ST (Supplementary Fig. 2d, e). The emergence of this subset of microglia in the TASTPM dorsal horn was not associated with signs of neurodegeneration since MAP2 expression was comparable to WT dorsal horn (Supplementary Fig. 2f, g). It is unlikely that CD11c and MHCII positive cells were monocytes or dendritic cells as we gated for CD45^low^ Ly6C^−^ CCR2^−^ cells (Supplementary Fig. 3), and the BBB of TASTPM mice is reported to be intact^16^. In addition, dendritic cells are defined as CD45^high^ populations and normally populate the meninges^29^, which we excluded from tissues before flow cytometry analysis.

So far, at day 5 K/BxN ST, our data indicate that in WT, CX3CR1^+^ DH microglia adopt a specific expression pattern, namely p-p38^+^, P2Y12^+^, CD68^+^ in association with K/BxN ST mechanical hypersensitivity and in TASTPM, CX3CR1^+^ DH microglia adopt a distinct amyloid-responsive expression pattern, namely P2Y12^−^, TREM2^+^, MerTK^+^, CD11c^+^, MHCII^+^, CD68^+^ which is associated with milder K/BxN ST allodynia. These considerations persuaded us to keep our focus on microglia at day 5 after K/BxN ST, and we characterised WT and TASTPM dorsal horn microglia transcriptional profile.

### WT K/BxN ST microglia have a unique transcriptional signature that is only partially present in TASTPM K/BxN ST

**Fig. 3:**
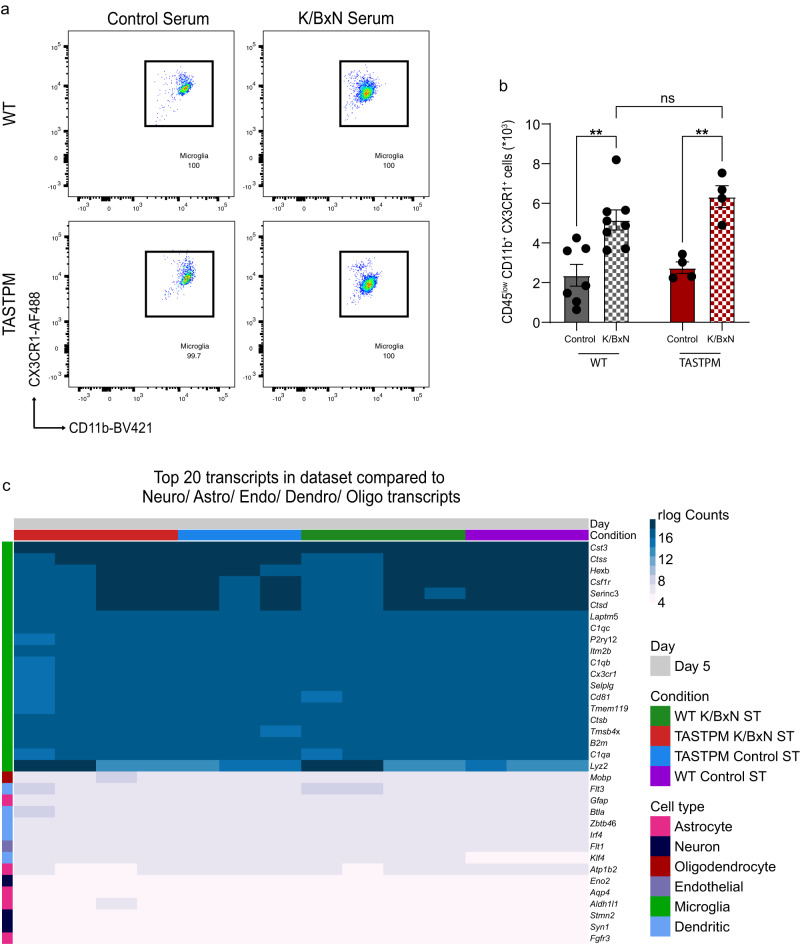
Dorsal horn microglia in WT and TASTPM K/BxN ST highly express homoeostatic genes. **a** Representative scatterplots of CD45^low^ CD11b^+^ CX3CR1^+^ DH microglia. **b** Quantification of microglia number in DH. Data represent mean ± SEM, *n* = 7 (WT Control), *n* = 8 (K/BxN ST), and *n* = 4 (TASTPM Control and K/BxN ST). **p < 0.01 compared to the same genotype Control ST. Two-way ANOVA, Tukey’s multiple comparisons test. **c** Top 20 expressed transcripts identified in microglia in DH. Minimal expression of non-microglial transcripts in the sorted cells. *n* = 3 (WT and TASTPM Control), *n* = 4 (WT and TASTPM K/BxN ST).

**Fig. 4:**
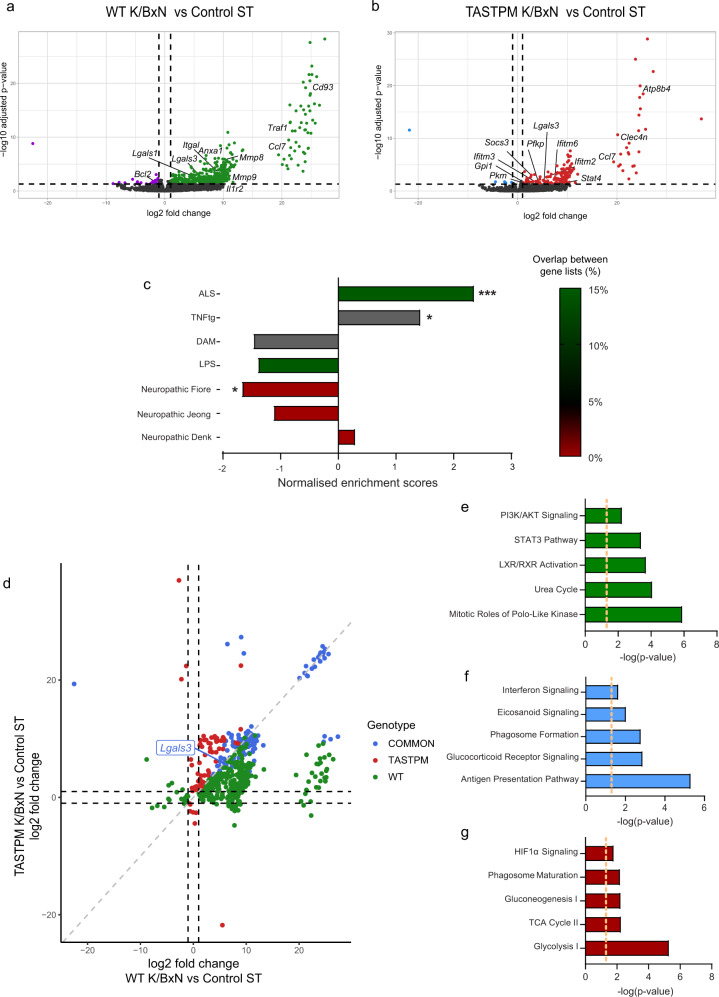
K/BxN ST is associated with the expression of a unique subset of transcripts in WT dorsal horn microglia that is only partially induced in TASTPM microglia. **a** Volcano plot showing differentially expressed genes in WT K/BxN ST versus Control ST DH microglia. The horizontal dashed line represents the FDR < 0.05 cutoff (two-sided Student’s *t*-test, Benjamini–Hochberg FDR). Upregulated transcripts (log_2_FC > 1 and FDR < 0.05) are marked with green. Downregulated transcripts (log_2_FC < −1 and FDR < 0.05) are marked with purple. **b** Volcano plot shows differentially expressed genes in TASTPM K/BxN ST versus Control ST microglia. Upregulated transcripts (log_2_FC > 1 and FDR < 0.05) are marked with red. Downregulated transcripts (log_2_FC < −1 and FDR < 0.05) are marked with blue. **c** Pairwise comparison of DH microglia in WT K/BxN ST with transcriptome data from mouse AD, ALS, RA and neuropathic pain models as well as LPS. High overlap (>10%) between datasets is marked with green, medium (3–10%) with grey, while minimal to no (0–3%) overlap is marked with red. Pairwise dataset overlap, normalised enrichment scores (NES), as well as FDR values were determined by GSEA analysis. The estimated probability that NES represents a false positive finding was calculated using an FDR cutoff of 5%. *FDR < 0.05, ***FDR < 0.001. **d** Four-way plot depicting DEGs (FDR < 0.05) that are TASTPM K/BxN-specific transcripts (red), common in WT and TASTPM K/BxN ST (blue), WT K/BxN-specific transcripts (green). **e**–**g** Top canonical pathways identified by IPA that are differentially regulated between TASTPM K/BxN-specific transcripts (red), WT and TASTPM K/BxN ST transcripts (blue), WT K/BxN-specific transcripts (green). *n* = 3 (WT and TASTPM Control), *n* = 4 (WT and TASTPM K/BxN ST).

Upon CNS injury or inflammatory insult, microglia shift from a homoeostatic role to a reactive state which is driven by prominent transcriptional changes^30,31^. Here, we evaluated WT and TASTPM microglia transcriptional profile in inflammatory arthritis nociception and performed bulk RNA-seq on microglia (CD45^low^, CD11b^+^, CX3CR1^+^) sorted from day 5 K/BxN ST dorsal horns, with a purity of >99% (Fig. 3a). In agreement with microgliosis detected by immunostaining, we found that CD45^low^ CD11b^+^ CX3CR1^+^ cell numbers were higher in K/BxN ST than control ST in both WT and TASTPM dorsal horns (Fig. 3a, b). In addition, 20 most highly expressed transcripts across all samples were typical microglia-specific transcripts (*Cx3cr1*, *Ctss*, *Hexb*, *Csf1r*, *P2ry12*, *Tmem119*, *C1qa*) (Fig. 3c), and no transcripts uniquely expressed by astrocytes, neurons, endothelial cells, oligodendrocytes, and dendritic cells were detected in any sample, confirming the purity of the microglia population sequenced (Fig. 3c). Differential gene expression (DE) analysis in WT microglia between control ST and K/BxN ST identified 483 differentially expressed genes (DEGs) (log_2_FC > 1, FDR < 0.05), of which 442 and 41 were up- and downregulated, respectively (Fig. 4a and Supplementary Dataset 1). Pathway analysis of differentially expressed genes using Gene Ontology (GO) revealed that ‘positive regulation of cytokine production’ (*Irak3*, *Il27ra*, *Il1r2*) and ‘G protein-coupled receptor signalling’ (*P2ry14*, *P2ry1*, *Rgs18*, *Gpr132*) were among the most highly enriched pathways (Supplementary Fig. 4a). Interestingly, in WT differentially expressed transcripts were linked to the TNF-α and IL-1β production pathways as well as responses to IFN-β pathway, all of which are known to regulate dorsal horn nociceptive mechanisms in the K/BxN ST model of inflammatory arthritis^9,32^. DE analysis in TASTPM between control ST and K/BxN ST revealed 194 and 8 up- and downregulated transcripts, respectively, which suggested that TASTPM DH microglia were less responsive than WT under K/BxN ST inflammatory allodynia (Fig. 4b and Supplementary Dataset 2). Pathway analysis revealed that ‘response to IFN-γ’ (*Ifitm2*, *Ifitm3*, *Ifitm6*), ‘phagocytosis’ (*Anxa1*, *Pparg*) and ‘NAD metabolic process’ (*Pfkp*, *Pkm*, *Gpi1*, *Aldh1b1*) were among the most highly enriched pathways (Supplementary Fig. 4b), all of which are associated with glycolytic activity and metabolic exhaustion in microglia in response to amyloid oligomers in AD^33^.

Following, to determine whether such a microglia transcriptional profile was uniquely instigated in the dorsal horn of WT in response to K/BxN ST inflammatory arthritis, we used gene set enrichment analysis (GSEA) and compared K/BxN ST microglia transcriptional profile with that of microglia in mouse models of neuropathic pain^34–36^, amyotrophic lateral sclerosis (ALS)^37^, lipopolysaccharide (LPS) stimulation^38^, RA thalamus^39^ and AD^25^. This analysis presented a significant enrichment of arthritis-specific microglia with spinal microglia in the SOD1 model of ALS and thalamic microglia in the TNFtg mouse model of RA (Fig. 4c). Furthermore, no significant enrichment could be detected with cerebellar microglia from LPS treated mice as well as disease-associated microglia (DAM) in the 5xFAD mice (Fig. 4c). Of note, negative or no correlation could be detected with microglial changes in models of neuropathic pain, suggesting that DH microglia in inflammatory pain acquire a distinct transcriptional signature (Fig. 4c). This result was further corroborated when comparing the overlap between gene datasets showing that less than 2% of DEGs in WT K/BxN ST was dysregulated in DH microglia in models of neuropathic pain (Fig. 4c). Interestingly, a set of 124 upregulated transcripts was identified in both WT and TASTPM K/BxN ST microglia (Fig. 4d and Supplementary Dataset 3). To understand better the physiological function of the shared set of transcripts, we used IPA canonical pathway analysis and observed that antigen presentation and phagocytosis were associated with these transcripts (Fig. 4e–g). Subsequent analysis with IPA on the WT- and TASTPM-specific transcripts uncovered significant activation of the STAT3 pathway and inhibition of PI3K/AKT signalling solely in WT K/BxN ST (Fig. 4e), phagosome formation in the transcriptionally commonly dysregulated in WT and TASTPM K/BxN ST (Fig. 4f) and activation of Glycolysis and the TCA cycle in TASTPM K/BxN ST (Fig. 4g).

These results highlight that in association with K/BxN inflammatory allodynia, WT microglia in the dorsal horn manifest a unique transcriptional profile, which is functionally related to cytokine pathways (IL-1β, TNF-α, IFN-β) and pro-inflammatory STAT3 pathway. In contrast, DH microglia in TASTPM show a response which features transcripts associated with increased glycolysis and dysregulated metabolism. Therefore, we subsequently explored which local cues could instigate the observed transcriptional profiles.

### TLR4 activation underlies transcriptional changes in dorsal horn microglia only in WT K/BxN

**Fig. 5:**
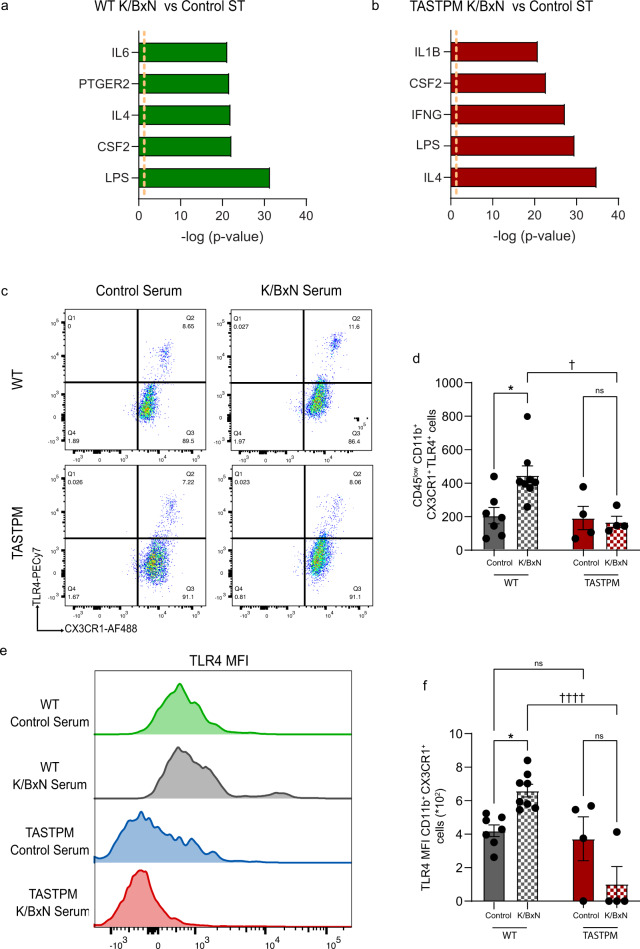
TLR4 mediates transcriptional changes in dorsal horn microglia in WT but not TASTPM K/BxN ST. **a** IPA upstream regulator analysis of differentially expressed genes (FDR < 0.05 and |log_2_FC| > 1) between WT K/BxN ST and WT Control ST. **b** IPA upstream regulator analysis of differentially expressed genes (FDR < 0.05 and |log2FC| > 1) between TASTPM K/BxN ST and TASTPM Control ST. *n* = 3 (WT and TASTPM Control), *n* = 4 (WT and TASTPM K/BxN ST). **c** Representative scatterplots of TLR4^+^ microglia. **d** Quantification of TLR4^+^ microglia in DH. **e** Mean fluorescence intensity (MFI) of TLR4 in DH microglia. **f** Quantification of TLR4 MFI. Data represent mean ± SEM, *n* = 7 (WT Control ST), *n* = 8 (WT K/BxN ST), and *n* = 4 (TASTPM Control and K/BxN ST). ns = not significant, *p < 0.05, ††††*p* < 0.0001. Two-way ANOVA, Tukey’s multiple comparisons test.

To investigate a mechanism responsible for the induction of a distinct transcriptional profile in K/BxN ST microglia, we performed annotated functional transcriptomics using the IPA Upstream regulator analysis. Our analysis predicted the pro-inflammatory endotoxin LPS as the most significant upstream regulator in WT K/BxN ST, while LPS was the second most significant upstream regulator after IL4 in TASTPM K/BxN ST (Fig. 5a, b). Thus, to validate bioinformatics analyses, we evaluated the microglial expression of LPS receptor Toll-like receptor 4 (TLR4) by flow cytometry, although *Tlr4* gene expression was not modulated in WT and TASTPM K/BxN ST at day 5 (Supplementary Fig. 5a). In WT microglia (CD45^low^ CD11b^+^ CX3CR1^+^ cells), we observed a higher proportion of TLR4^+^ cells and higher TLR4 expression at the single-cell level in K/BxN ST compared to control ST (Fig. 5c–f). In contrast, in TASTPM, neither number of TLR4^+^ microglia nor TLR4 cellular expression was altered in K/BxN ST compared to control ST (Fig. 5c–f). Specifically, TLR4 expression was significantly lower in TASTPM K/BxN ST microglia than WT K/BxN ST (Fig. 5c–f), suggesting that molecules other than TLR4 might be driving changes in transcriptional profile in TASTPM DH microglia. Relevant to WT transcriptional profile data, TLR4 plays a critical role in K/BxN ST nociceptive mechanisms since both TLR4 knock-out mice and intrathecal injection of TLR4 antagonists are associated with reversal of K/BxN ST allodynia^13,40^. Consistently, we observed that an intrathecal injection of LPS-RS (10 μg/5 μl/mouse; penta-acylated LPS from *Rhodobacter sphaeroides* that acts as a TLR4 antagonist^41^ reversed K/BxN ST allodynia to a comparable extent in males and females (Supplementary Fig. 5b).

Altogether, our data in WT demonstrated an association between K/BxN ST allodynia and upregulation of microglial TLR4, which we predicted to drive the observed transcriptional profile in DH microglia. Consistently, in TASTPM, milder K/BxN ST allodynia was associated with lower TLR4 expression and altered transcriptional profile in DH microglia. Thus, our next step was to investigate whether endogenous TLR4 ligands were dysregulated in WT and TASTPM microglia.

### In K/BxN ST, Gal-3 is dramatically upregulated in microglia and increased in primary afferent terminals

**Fig. 6:**
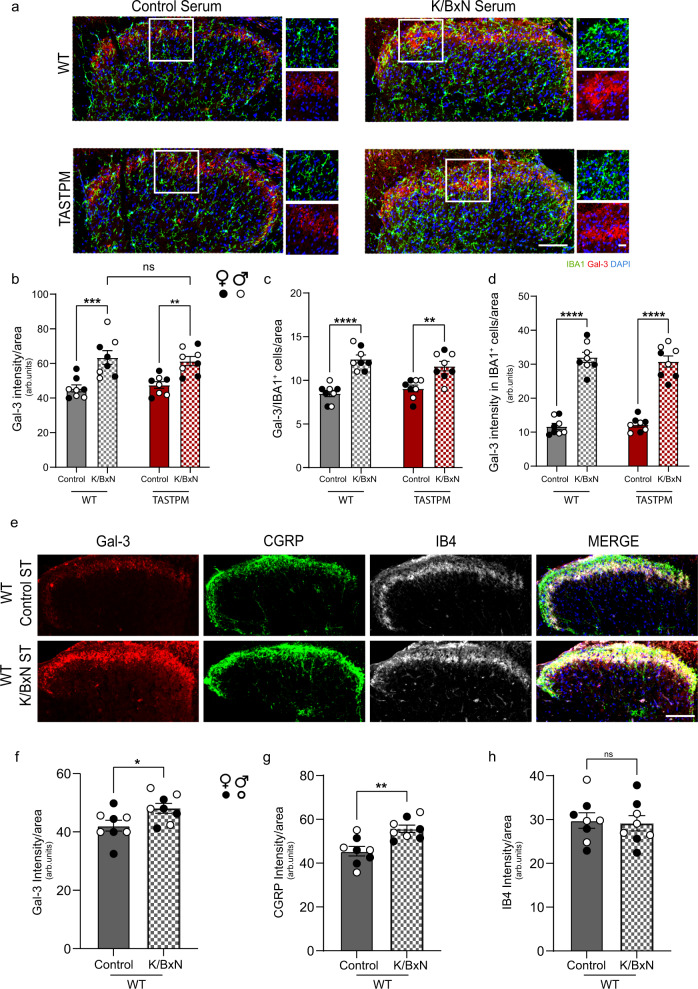
Gal-3 is upregulated in dorsal horn microglia and primary afferent terminals in WT and TASTPM K/BxN ST. **a** Representative images of Gal-3 and IBA1 in DH. Scale bar, 100 and 20 μm in insets. **b** Quantification of Gal-3 intensity in the dorsal lumbar spinal cord of WT and TASTPM Control and K/BxN ST. **c** Quantification of Gal-3/IBA1^+^ cell number in DH. **d** Quantification of Gal-3 intensity in IBA1^+^ cells in DH. Data represent mean ± SEM, *n* = 8 mice per group (4 per sex). ns not significant, *p < 0.05, **p < 0.01, ***p < 0.001, ****p < 0.0001. Two-way ANOVA, Tukey’s multiple comparisons test. **e** Representative images of Gal-3, CGRP and IB4 in WT DH. Scale bar, 100 μm. **f**–**h** Quantification of Gal-3, CGRP and IB4 intensity in DH. *p = 0.0327, **p = 0.0023. Unpaired Student’s *t*-test, two-tailed. Data represent mean ± SEM, *n* = 8 mice per group (4 per sex).

The crucial role of the TLR4 pathway in inflammatory nociception is supported by evidence, including ours, that spinal cord blockade of either TLR4 signalling pathway or TLR4 endogenous ligands, such as high mobility group box-1 protein (HMGB1), can alleviate and prevent pain-like behaviour^13,40^. Thus, to assess a possible involvement of microglia-derived endogenous TLR4 ligands in K/BxN ST nociception, we identified differentially expressed transcripts that encode proteins known to bind TLR4. Specifically, differential gene expression analysis showed upregulation of *Hmgb2* only in WT K/BxN ST, *Hmgb3* in TASTPM K/BxN ST and *Lgals3* in DH microglia in both conditions. We engaged with *Lgals3*, which encodes for galectin-3 (Gal-3), a lactose-binding lectin, for the following two reasons. First, Gal-3 is normally expressed at low levels by microglia in homoeostasis and promotes cytokine release through activation of microglial TLR4 in various neurological disorders^42^. Second, Gal-3 is upregulated in disease-associated microglia in neurological disorders^43–45^ and binds microglial TREM2^46^ that we found was expressed at higher levels in TASTPM than WT DH microglia and upregulated in TASTPM K/BxN ST microglia. Therefore, to validate whether the expression of *Lgals3* is reflected in changes at the protein level, we assessed Gal-3 immunostaining. In WT and TASTPM (male and female), we observed distinct Gal-3 fluorescence intensity in superficial laminae in the control ST dorsal horn, which increased in K/BxN ST (Fig. 6a, b). Under control conditions, Gal-3 was not expressed by dorsal horn neurons and was seen in a few astrocytes and microglia (Supplementary Fig. 6a–c). Then, in agreement with our DE analysis, we observed that Gal-3 was upregulated by about 3 folds in microglia (IBA1^+^ cells) both in WT and TASTPM K/BxN ST dorsal horns (Fig. 6c, d). Furthermore, to assess the potential association between increased Gal-3 and microglia numbers, we investigated whether Gal-3 is associated with increased microglial proliferation in vitro. In agreement with the lack of Gal-3 effect on BV2 microglia cell cycle^42^, we observed no significant proliferation of primary microglia following incubation with Gal-3 at 300 nM (Supplementary Fig. 6d, e), a concentration that induces proliferation of fibroblasts^47^.

Since Gal-3 expression was confined to the superficial laminae of the dorsal horn under control ST conditions, we examined whether Gal-3 could be found in primary afferent terminals (Fig. 6e, f). We observed that in WT control ST dorsal horn, Gal-3 was mainly expressed in non-peptidergic (IB4^+^) terminals in lamina II but not in peptidergic (CGRP^+^) terminals (Fig. 6e and Supplementary Fig. 7a–c)_._ In addition, in WT K/BxN ST dorsal horn, Gal-3, CGRP but not IB4 immunostainings were increased (Fig. 6e–h), and Gal-3 was found to colocalise with IB4^+^ but also with CGRP^+^ terminals (Fig. 6e and Supplementary Fig. 7a–c). As expected^48^, we found some CGRP colocalization with IB4, which remained unchanged in K/BxN ST dorsal horns (Supplementary Fig. 7a, b). Altogether, these data demonstrate that under control conditions, Gal-3 is expressed by nociceptive afferent terminals in the dorsal horn. In K/BxN ST inflammatory arthritis, there is an increased level of Gal-3 in the superficial laminae, and more Gal-3 is expressed by microglia in the vicinity of nociceptive terminals.

### Gal-3 is expressed and released by a subtype of DRG nociceptors in control ST and expression pattern and release change in K/BxN ST

**Fig. 7:**
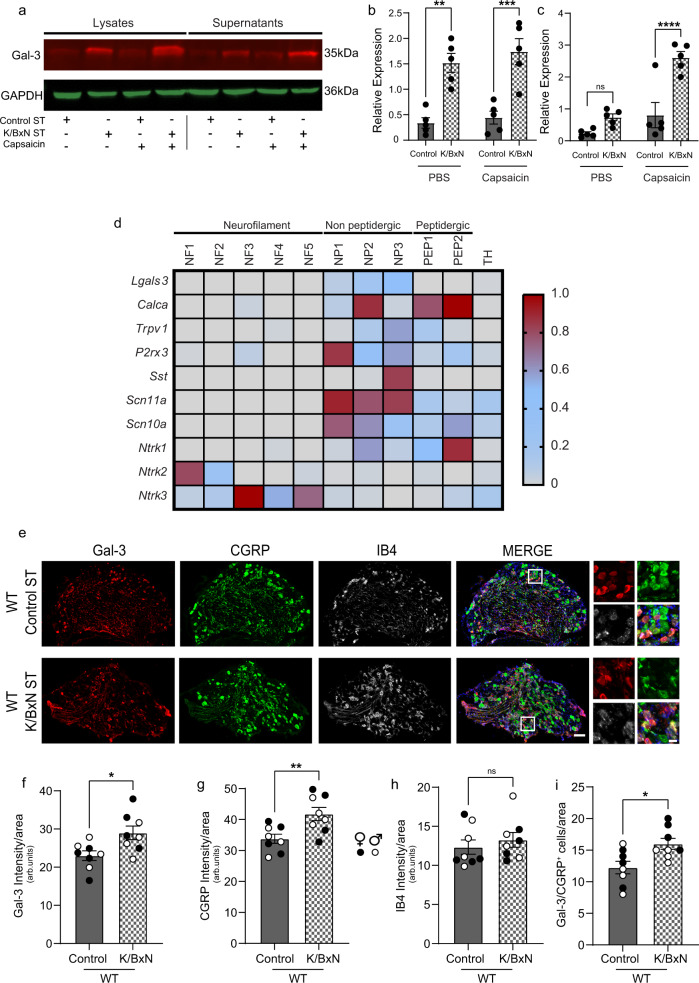
Gal-3 is expressed and released by a subtype of DRG nociceptors in control ST and Gal-3 expression pattern changes in K/BxN ST. **a**–**c** Western blot and quantification of Gal-3 in synaptosome lysates (**a**, **b**) and supernatants (**a**, **c**) after incubation with capsaicin (1 μM for 3 h). Data represent mean ± SEM, *n* = 5 mice per group. ns = not significant, *p < 0.05, two-way ANOVA, Tukey’s multiple comparisons test. These experiments were repeated three times. **d** Expression of *Lgals3* and subgroup markers across sensory neuron subsets from published scRNA-seq data. **e** Representative images of Gal-3, CGRP and IB4 in lumbar DRG. Scale bar, 100 and 20 μm in the inset. **f**–**h** Quantification of Gal-3, CGRP and IB4 intensity in lumbar DRG. **i** Quantification of Gal-3^+^ CGRP^+^ neurons in lumbar DRG of WT Control and K/BxN ST. Data represent mean ± SEM, *n* = 8 mice per group (4 per sex). *p < 0.05, **p = 0.008. Unpaired Student’s *t*-test, two-tailed.

From a functional perspective, these immunostaining data suggest the intriguing possibility that Gal-3 is released by primary afferent terminals and such a release would increase in K/BxN ST dorsal horn. To test this hypothesis, we quantified Gal-3 levels in WT and TASTPM dorsal horn synaptosomes incubated with capsaicin, an in vitro model of noxious-like stimulation of isolated nerve endings. We observed that Gal-3 protein was detectable in synaptosome lysates of WT and TASTPM control ST dorsal horn, and levels were upregulated in lysates obtained from K/BxN ST dorsal horn (Fig. 7a–c Supplementary Fig. 8a–c). Incubation of synaptosomes with capsaicin (1 μM for 3 h) did not alter Gal-3 levels in WT synaptosome lysates (Fig. 7a, b). However, Gal-3 levels were significantly increased in the supernatants of control ST and K/BxN ST synaptosomes following capsaicin incubation in both WT and TASTPM (Fig. 7a, c and Supplementary Fig. 8a, c). Notably, Gal-3 was not detected in the extracellular vesicle fraction of synaptosome supernatants (Supplementary Fig. 8d).

Since TRPV1 receptors for capsaicin are expressed by primary afferent terminals in the dorsal horn^49^, these data suggest that in both WT and TASTPM K/BxN ST dorsal horn, nociceptive afferent terminals release Gal-3 with activity. To examine the subtype of sensory neurons that express *Lgals3* in the cell bodies and would likely release Gal-3 in the dorsal spinal cord, we mined a publicly available single-cell RNA-sequencing (scRNA-seq) dataset of mouse DRG neurons^50^. Our analysis revealed that *Lgals3* expression was higher in the subset of non-peptidergic 3 (NP3) nociceptors expressing *Trpv1*, *P2rx3*, *Sst*, and *Scn11a* (Nav1.9). Furthermore, *Lgals3* could also be detected in a group of NP2 nociceptors expressing *Calca* (CGRP), *Scn10a* (Nav1.8), *Scn11a* (Nav1.9) and *Ntrk1* (TrkA) (Fig. 7d). Next, we performed immunostaining of DRG neurons to validate the scRNA-seq data. We detected Gal-3 protein expression in neurons with small cell bodies in WT control ST and upregulation in K/BxN ST (Fig. 7e, f). Similarly, the neuropeptide CGRP immunostaining was upregulated in WT K/BxN ST DRG (Fig. 7g), whilst IB4 intensity was not significantly altered (Fig. 7h). Gal-3 was found predominantly in IB4 neurons under control ST conditions, but it was also expressed in CGRP peptidergic neurons, especially in K/BxN ST DRG (Fig. 7i and Supplementary Fig. 9a–c). Altogether, this immunohistochemical evidence in WT shows that Gal-3 is expressed in DRG neurons which terminate in the superficial dorsal horn of the spinal cord, and in inflammatory arthritis, Gal-3 is also expressed by a small number of peptidergic (CGRP^+^) neurons.

So far, our approach combining transcriptomics and molecular techniques had led to the discovery of a distinct neuron-to-microglia communication pathway in the context of inflammatory pain. Specifically, in WT K/BxN ST dorsal horn Gal-3 is upregulated in primary afferent fibre terminals, released in response to noxious stimulation and therefore it is likely able to activate TLR4 receptor expressed by microglia to promote cytokine release. In turn, TLR4-mediated release of pro-inflammatory cytokines would contribute to the sensitization of nociceptive DH neurons and result in behavioural allodynia. We presumed that this pathway might be actively involved with K/BxN-induced allodynia in both WT and TASTPM; hence, to verify its functional relevance, we tested the effect of a Gal-3 inhibitor on K/BxN ST allodynia in WT and TASTPM mice.

### Gal-3 exerts a pro-nociceptive effect via TLR4, and Gal-3 inhibitor attenuates K/BxN ST allodynia in WT but not TASTPM mice

**Fig. 8:**
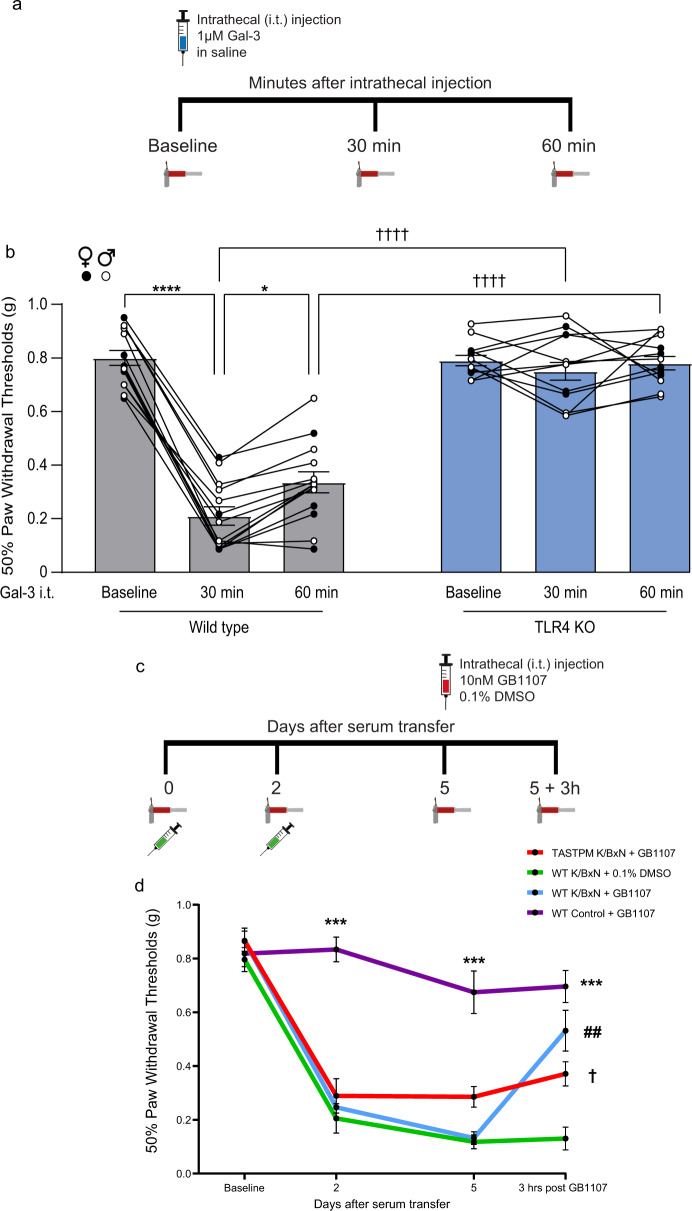
Intrathecal Gal-3 exerts a pro-nociceptive effect that requires TLR4 expression, and Gal-3 inhibitor attenuates K/BxN ST allodynia in WT but not TASTPM mice. **a** Gal-3 (170 ng/ 5 μl; 1 μM) intrathecal injection in WT and TLR4 KO followed by behaviour at 30 and 60 min after injection. **b** Exogenous Gal-3-induced mechanical hypersensitivity in WT but not TLR4 KO mice. Data represent mean ± SEM, *n* = 8 (TLR4 KO, 4 per sex), *n* = 12 (WT, 6 per sex). *p < 0.05, ****p < 0.0001, ††††*p* < 0.0001. Two-way RM ANOVA, Tukey’s multiple comparisons test. **c** Intrathecal injections of GB1107 (10 nM) or 0.1% DMSO performed at Day 5 K/BxN and behaviour at 3 h after injection. **d** Reversal of mechanical hypersensitivity by GB1107 in WT but not TASTPM K/BxN ST. Data represent mean ± SEM, *n* = 8 mice per group. ***p < 0.001 WT K/BxN ST vs. WT Control ST, †*p* < 0.05 TASTPM K/BxN ST vs. same-day WT K/BxN ST, ##*p* < 0.01. GB1107− WT KBxN ST vs. DMSO− WT K/BxN ST, two-way RM ANOVA, Tukey’s multiple comparisons test.

Since Gal-3 exerts pro-nociceptive effects when injected intrathecally in naïve mice^51^, we tested whether such an effect is mediated by spinal TLR4 receptors. We first confirmed that intrathecal delivery of Gal-3, but not heat-inactivated Gal-3, was pro-allodynic in male and female WT (Fig. 8a, b and Supplementary Fig. 10a). We used a dose of 170 ng/5 μl/mouse to match Gal-3 affinity for TLR4 (Kd =1 μM)^42^. Then we observed that the pro-nociceptive effect of Gal-3 was significantly attenuated in male and female TLR4 knock-out (KO) mice (Fig. 8b), suggesting that exogenous Gal-3-induced nociception requires TLR4 expression. Subsequently, we evaluated whether endogenous Gal-3 contributes to allodynia in inflammatory arthritis and injected a Gal-3 inhibitor, GB1107 (10 nM), intrathecally into male and female WT mice at day 5 after K/BxN ST (Fig. 8c). We observed that at 3 h after injection, the GB1107-treated group thresholds were significantly higher than vehicle control, although paw swelling was unaltered (Supplementary Fig. 10b, c), indicating a reversal of allodynia. Finally, having established that GB1107 reversal of allodynia was sex-independent, we assessed the effect of the Gal-3 inhibitor in TASTPM male mice at day 5 after K/BxN ST (Fig. 8d). We confirmed that WT had developed mechanical allodynia and TASTPM less severe allodynia (Fig. 8d). However, in TASTPM, we observed that thresholds before and after injection of GB1107 were unaltered indicating no significant effect on K/BxN ST allodynia.

**Fig. 9:**
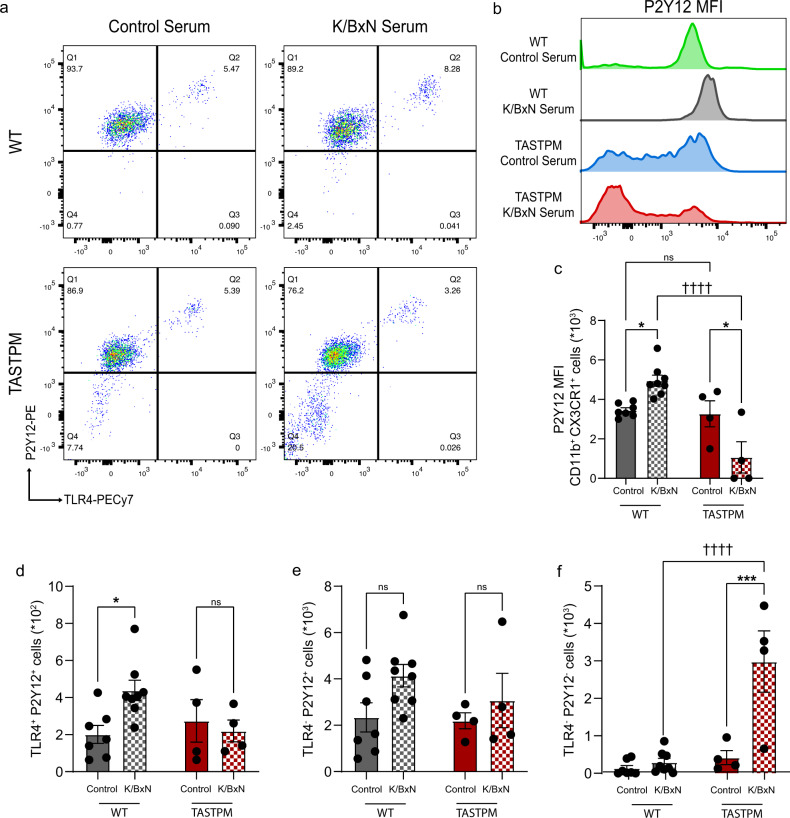
A subset of microglia emerges in the dorsal horn of TASTPM K/BxN ST. **a** Representative scatterplots of CD45^low^ CD11b^+^ CX3CR1^+^ microglia gated on P2Y12 and TLR4. **b** Mean fluorescence intensity (MFI) plots of P2Y12 in microglia. **c** Quantification of P2Y12 MFI. **d**–**f** Quantification of P2Y12^+^/TLR4^+^, P2Y12^+^/TLR4^−^, P2Y12^−^/TLR4^−^ microglia in dorsal horns. Data represent mean ± SEM, *n* = 7 (WT Control ST), *n* = 8 (WT K/BxN ST), and *n* = 4 (TASTPM Control and K/BxN ST). ns not significant, *p < 0.05, ***p < 0.001, ††††*p* < 0.0001. Two-way ANOVA, Tukey’s multiple comparisons test.

To determine molecular mechanisms underlying the differential responses of WT and TASTPM K/BxN ST mice to GB1107, we performed flow cytometry analysis of TLR4 expression in DH microglia. This analysis revealed that similarly to P2Y12, TLR4 was upregulated in microglia of WT K/BxN ST and downregulated in TASTPM K/BxN ST (Fig. 9a–c). Specifically, the WT microglia pool consisted of a P2Y12^+^/TLR4^+^ population that was increased in K/BxN ST (Fig. 9d) and a P2Y12^+^/TLR4^−^ population that did not change in K/BxN ST (Fig. 9e). Interestingly, TASTPM microglia pools were more heterogeneous, as P2Y12^+^/TLR4^+^ cells were unchanged (Fig. 9d) and a subset of numerous P2Y12^−^/TLR4^−^ cells emerged (Fig. 9f).

These data indicate that in WT dorsal horn, microglia express TLR4 receptors which can be activated by Gal-3 released by noxious fibre terminals in K/BxN ST and facilitate nociceptive neuron activation. Differently, a cluster of P2Y12^−^/TLR4^−^ microglia emerges in the TASTPM K/BxN ST, highlighting that Gal-3 released by nociceptive fibres in TASTPM dorsal horn could not induce TLR4-mediated activation of microglia. The lack of TLR4 signalling in the TASTPM microglia subset would account for the Gal-3 inhibitor’s inability to reverse allodynia in TASTPM. Furthermore, the emergence of microglia lacking P2Y12 and TLR4 and expressing CD11c in TASTPM K/BxN ST correlates with the development of weaker allodynia. Whether or not this microglia population explains the attenuation of allodynia at day 5 KBxN ST is unclear, but this seems highly probable as the absence of P2Y12 and TLR4 renders these cells unresponsive to both ATP/ADP and Gal-3, and the emergence of a CD11c^+^ microglia subset is associated with resolution of neuropathic allodynia^18^.

## Discussion

This study provides evidence for a neuro-immune pathway that mediates sensory neuron-to-microglia communication in the dorsal horn of the spinal cord under inflammatory arthritis pain conditions. We show the involvement of microglia in the development of inflammatory pain and define molecular cues that shape microglial responses in-vivo and ex-vivo. Specifically, we provide evidence that exogenous Gal-3 exerts a sex-independent pro-nociceptive effect in WT but not TLR4 KO, and endogenous Gal-3 contributes to inflammatory arthritis allodynia in male and female WT. Gal-3 is expressed and released by nociceptive neurons in the dorsal horn, where microglia acquire a TLR4-dependent transcriptional profile. Consistent with our data, intrathecally injected Gal-3 is reported to exert pro-nociceptive actions in the spinal cord, and anti-Gal-3 antibodies reverse herpetic allodynia^51^. However, in the postherpetic neuralgia dorsal horn, the source of Gal-3 are microglia and infiltrating macrophages^51^. Instead, in inflammatory arthritis, we provide unequivocal evidence that Gal-3 is expressed by non-peptidergic and peptidergic sensory neurons and upregulated by microglia. In support of our findings, nociceptive neurons express *Lgals3*, gene encoding Gal-3, as established in recent scRNA-seq analysis in mouse DRG^50^ and spatial transcriptomics of human DRG^52^. Furthermore, older evidence in cultured DRG shows that Gal-3 expression can be increased by a nerve-growth factor (NGF)^53^, hinting that increased NGF levels in inflamed joints might be driving the upregulation of Gal-3 in TrkA/CGRP neurons. Our most critical observation is that Gal-3 is released by capsaicin in dorsal horn synaptosomes, providing evidence for an activity-dependent release of Gal-3 by nociceptors. Gal-3 lacks signal sequence for transport into the endoplasmic reticulum; therefore, it is not packaged into extracellular vesicles and bypasses classical Golgi secretory route^54,55^, and indeed, we observed no presence of Gal-3 synaptosome-derived vesicles. We propose that Gal-3 is released in free form in the dorsal horn, and extracellular Gal-3 induces the release of pro-inflammatory cytokines through activation of TLR4 in microglia^42^.

The role of microglia in neuropathic pain is well-established, as detailed transcriptional characterisation and inhibition paradigms highlight their function in the development, maintenance and resolution of neuropathic pain^56,57^. More specifically, at the onset of neuropathic pain, dorsal horn microglia respond to sensory neuron-derived CSF1, upregulate transcripts such as *Ctss*, *Cx3cr1*, *Itgam*, and *Bdnf* and facilitate nociceptive mechanisms^22,58^. Then, during neuropathic pain resolution, an emergent CD11c^+^ microglial pool contributes to the resolution of nociception by releasing IGF-1^18^. Nonetheless, much less progress has been made in delineating the mechanism through which sensory neurons interact with microglia in inflammatory pain. Here, we show that in arthritis pain, dorsal horn microglia acquire a unique transcriptional profile that is driven by TLR4 upregulation and is enriched in pathways regulating the production and effect of IL-1β, TNF-α and IFN-β, which have been reported to regulate nociception in the K/BxN ST model^13,32^. Indeed, we confirmed a TLR4 receptor pro-allodynic role in K/BxN ST inflammatory arthritis^13^ as intrathecal delivery of LPS-RS reversed inflammatory allodynia in a sex-independent fashion. Furthermore, our results also highlight that dorsal horn microglia significantly upregulate P2Y12, which is responsible for sensing neuronal activity through diffusible factors such as ATP/ADP^59^ and is required for the development of neuropathic^60,61^ and acute inflammatory pain^62^. Intriguingly, the upregulation of P2Y12 in K/BxN ST hints at increased microglial dynamics that will result in perturbed interaction between microglia, neurons^63^ and the vasculature^64,65^. In turn, microglia respond to increased activity at the first sensory synapse, which is associated with inflammatory arthritis through upregulation of CD68 and possible degradation of perineuronal nets^66^ and synapses^67^, and they overall contribute to nociceptive mechanisms by perturbing neural circuit functionality.

In addition, the indispensable role of microglia in the development of inflammatory arthritis pain is corroborated by our studies using the TASTPM mouse model of AD. Our results show that prior to amyloid plaque deposition in the spinal cord at 12 months of age^15^, and after the induction of arthritis, microglia express markers, such as CD68, TREM2, CD11c, MerTK, MHCII, and downregulate P2Y12, all of which are upregulated in response to rising levels of amyloid-β and amyloid plaques^25,44^. This pattern is also mirrored at the transcriptional level, as TASTPM microglia express transcripts involved in glycolysis, in line with evidence of amyloid-β-induced metabolic defects in microglia^33^, as well as interferon-response pathways^68^. These results suggest that in the spinal cord, similar to the hippocampus of two-month-old TASTPM^69^, rising levels of oligomeric soluble amyloid-β can account for microglia priming and desensitisation. Indeed, the distinct amyloid-responsive expression pattern is associated with the mitigated development of inflammatory allodynia in TASTPM. Similarly, we previously reported that TASTPM shows attenuated pain-like behaviour in a model of osteoarthritis pain^70^. The attenuated hypersensitivity in TASTPM is closely linked with the emergence of a subset of microglia that are not responsive to ATP/ADP released by excited neurons as they lack P2Y12 expression^59^. Relevantly, central P2Y12 antagonists alleviate inflammatory pain^61^ and P2ry12^−/−^ mice show similar insensitivity to noxious hot stimulation as the TASTPM^15,61^. Moreover, the TREM2^+^ P2Y12^−^ microglia subgroup does not express TLR4, which renders microglia specifically unresponsive to Gal-3 released from nociceptors in inflammatory arthritis in TASTPM. In line with this observation, the administration of Gal-3 inhibitors did not reverse nociception in TASTPM mice. This finding is consistent with evidence that TREM2 and TLR4 are oppositely regulated in cultured microglia and mouse brain^38,71,72^. Indeed, in the context of amyloidosis, Gal-3 can act as an endogenous ligand for TREM2 (K_d_ = 450 nM)^46^, with higher affinity than for TLR4 (K_d_ = 1 μM)^42^, suggesting that in the context of TASTPM inflammatory arthritis pain, Gal-3 might bind TREM2 as TLR4 is significantly downregulated. It is tempting to speculate that the Gal-3 effect on behaviour might be mediated by the activation of the TREM2 signalling pathway, which facilitates the expression of anti-inflammatory transcripts resembling the IL4 pathway through the activation of STAT6^71^. Although we cannot exclude a pro-nociceptive TREM2/DAP12 activation as suggested in neuropathic conditions^73,74^, such a role in inflammatory arthritis pain requires further investigation.

**Fig. 10:**
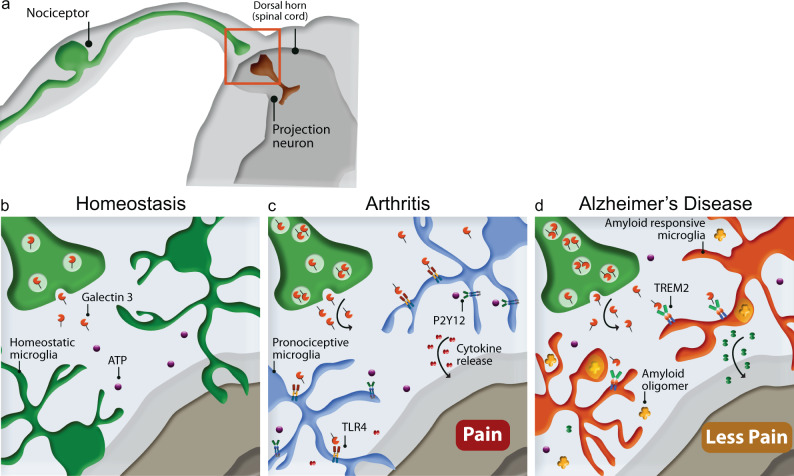
Attenuated inflammatory arthritis pain in TASTPM is caused by microglia that are insensitive to Gal-3. **a** Illustration of the superficial laminae of the dorsal horn of the spinal cord, where nociceptor terminals synapse on laminae I projection neurons. **b** In homoeostasis, microglia constantly survey the parenchyma by responding to cues in their microenvironment. **c** During inflammatory arthritis, TLR4^+^ P2Y12^+^ microglia respond to neuron-derived Gal-3 and ATP by releasing pro-inflammatory mediators that lead to sensitisation. **d** In TASTPM K/BxN ST, a subset of phagocytic TREM2^+^ TLR4^−^ P2Y12^−^ microglia emerges and contributes to attenuation of allodynia.

Overall, our data identify a mechanism through which nociceptors respond to joint inflammation and establish nociception through the activation of microglia. We suggest that sensory neuron-derived Gal-3, swiftly joined by Gal-3 derived from activated microglia, activates microglial TLR4 and promotes nociceptive signalling via the release of cytokines in the dorsal horn (Fig. 10a, b). Intriguingly, our data support the proposal that microglia mediate the Gal-3 effect as in the spinal cords of the TASTPM mouse model of AD, the emergence of a subset of microglia devoid of TLR4 and P2Y12 is associated with milder inflammatory nociception (Fig. 10c) and lack of antinociceptive effect of a Gal-3 inhibitor.

## Methods

### Animals

All procedures were performed under personal and project licences granted by the UK Home Office (Project Licence PC4DEDAC9 to MM) and complied with the United Kingdom Animal Scientific Procedures Act 1986. Approval for studies was provided by King’s Animal Welfare and Ethical Review Body, London, United Kingdom. Unless specified, all experiments were performed on 6-month-old adult male and female C57BL/6 WT and TASTPM mice weighing approximately 30–35 g. TASTPM mice were generated using TAS10 transgenic mice expressing the Swedish mutation in the human amyloid precursor protein (APP) and TPM mice expressing the human PSEN1 with the M146V mutation. Both transgenes are driven by the murine Thy-1 promoter^75^. Briefly, TAS10 mice were generated and backcrossed onto a pure C57BL/6 background before being crossed with TPM mice to produce heterozygous double mutant TASTPM mice (GlaxoSmithKline). Age- and sex-matched WT C57BL/6J controls were generated by backcrossing heterozygote TASTPM mice to produce mice with the same genetic background and no human transgenes. TLR4 KO mice on C57/BL6 background were kindly provided by Dr S. Akira, Osaka, Japan. All animals were housed in the Biological Services Unit, King’s College London, maintained at room temperature with a 12-h light/dark cycle, in groups of up to five per standard cage, with access to food and water ad libitum.

### Induction of the K/BxN serum transfer model

K/BxN serum was obtained as previously described^76^. Briefly, NOD/Shiltj mice were crossed to KRN transgenic mice to generate offspring (K/BxN mice) expressing both the T cell receptor (TCR) transgene KRN and the MHC class II molecule Ag7. These mice spontaneously develop severe inflammatory arthritis, and serum was collected from K/BxN mice at 10 weeks of age (provided by Dr Dianne Cooper, Queen Mary University of London). Arthritis was induced with 100 μl intraperitoneal (i.p.) injection of arthritogenic serum on days 0 and 2 (total volume 200 μl) in 6-month-old naïve WT and TASTPM mice. Control mice received equal volume injections containing pooled sera from KRN/C57 mice. Clinical signs of arthritis were evaluated using a 12-point scoring system^76^. Each limb was scored separately; 0 to 3 points per limb with the following criteria: 0—no sign of redness/swelling; 1—redness/swelling observed in either ankle/wrist, pad, or any of the digits; 2—redness/swelling in two regions; and 3—redness/swelling seen in all limb regions. Scores for all four limbs were combined to give a total score with a maximum score of 12 per animal.

### Behavioural testing

Hind paw mechanical withdrawal thresholds were assessed by applying a series of calibrated von Frey filaments (0.02–1.0 g; North Coast Medical) to the plantar surface of the hind paw. On each day of testing, animals were habituated for 30 min before the application of the initial 0.07-g filament. Filaments were then applied at increasing intensity until a withdrawal response was achieved or the application of 1.0-g filament failed to elicit a withdrawal response. Fifty per cent paw withdrawal threshold was calculated using the Dixon up and down method^77^. Briefly, if a withdrawal response to a stimulus was established, the paw was retested with the next lowest intensity filament below stimuli that elicited a withdrawal until no withdrawal occurred. After that, stimuli with ascending force filaments were applied until a response was observed. Fifty per cent withdrawal threshold calculation by the Dixon method requires a minimum of five, up to a maximum of nine filament applications. From the resulting pattern of responses, the 50% withdrawal threshold was interpolated^78^. Three sets of baseline measurements were made prior to the induction of inflammatory arthritis. The experimenter was blinded to treatment groups.

### Intrathecal injections

Under isoflurane anaesthesia, Gal-3 (1 μM) (mouse recombinant Galectin 3, 1197-GA R&D System), heat-inactivated Gal-3 (1 μM, at 80 °C for 1 h), GB1107 (10 nM), LPS-RS (10 μg) (tlrl-prslps, InvivoGen, Toulouse, France) were intrathecally injected into the mouse lumbar region (between the L4 and L5 vertebrae) of the spine using a 30G needle.

### Immunohistochemistry

For immunohistochemistry in perfuse-fixed tissue, mice were deeply anaesthetised by intraperitoneal injection of pentobarbital (Pentoject®, York, UK) and transcardially perfused with 0.9% saline solution followed by 4% paraformaldehyde (VWR Chemicals, Leicestershire, United Kingdom) in 0.1 M phosphate-buffered saline (PBS). After fixation, L3, L4, and L5 DRGs, lumbar spinal cord and brains were dissected, cut using a cryostat (Bright Instruments, Luton, United Kingdom) and mounted onto Superfrost Plus microscope slides (Thermo-Scientific, Leicestershire, United Kingdom). Sections were then blocked with 3% bovine serum albumin (BSA) (Sigma-Aldrich, Dorset, United Kingdom) for 1 h, then incubated overnight with antibodies against c-Fos (1:200, #2250, Cell Signaling), IBA1 (1:1000, Wako Chemicals; 1:300, ab5076, Abcam, Cambridge, United Kingdom), p-p38 (1:100, #9211, Cell Signaling), CD68 (1:200, ab125212, Abcam, Cambridge, United Kingdom), TREM2 (1:300, AF1729, R&D System), Gal-3 (1:300, AF1197, R&D System), CGRP (1:1000, #14959, Cell Signaling), IB4 (1:1000, #13250, Thermo Fisher Scientific), Neu-N (1:1000, #12943, Cell Signaling), GFAP (1:1000, Dako Cytomation, Glostrup, Denmark), MAP2 (1:2000, ab183830, Abcam, Cambridge, United Kingdom), CD11c (1:500, #97585, Cell Signaling). Slides are then washed three times for 10 min in 0.1% PBS-T followed by incubation for 1 h at room temperature in the appropriate secondary antibodies (Alexa Fluor 488-,546- or 647- conjugated antibody, 1:1000; Invitrogen, Paisley, United Kingdom). The specificity of immunoreactivity was confirmed by omitting the primary antibody. Images for immunofluorescence analysis were captured with a Zeiss LSM710 confocal microscope (Zeiss) and analysed using ImageJ (1.50i, Wayne Rasband, National Institutes of Health, USA). At least three sections from three mice per group were analysed. Quantitative assessment of immunofluorescence intensity and cell number was performed in ImageJ using three boxes of 4 × 10^4^ μm^2^ each, which were placed onto lateral, central, and medial dorsal horn laminae I-II. Colocalization heatmaps were generated using the Colocalization Colormap plugin (https://sites.google.com/site/colocalizationcolormap/home), and Pearson’s correlation coefficients were calculated using JACoP plugin on ImageJ^79^.

### Primary microglia in culture

Microglia were isolated from adult mice brains following the protocol by Bordt et al.^80^. Cells were seeded at 1 × 10^5^ per coverslip and left maturing for 5 days in 10% FBS, 1% P/S DMEM/F12 media containing M-CSF (100 ng/ml). Matured microglia were starved overnight in 1% FBS, 1% P/S media and then incubated with Gal-3 (300 nM) or GM-CSF (20 ng/ml) to induce proliferation similar to Dikmen et al.^81^ for 48 h in the presence of EdU dye. EdU staining was performed following the manufacturer’s guidance (Invitrogen, C10637).

### Microglia isolation

To isolate cells for bulk RNA-seq, mice were euthanized with pentobarbital and were subsequently transcardially perfused using ice-cold PBS 1x and the brain and spinal cord were quickly dissected after removal of the meninges and placed in PBS on ice. Microglia/myeloid cell extraction was carried out following a published protocol^82^. The whole procedure was done on ice with cold buffers containing RNase inhibitors (N2615, Promega). Briefly, the tissue was dounce homogenised in ice-cold PBS with loose and tight pestles. The cell suspension was then transferred through a pre-wet 70-μm cell strainer (BD Falcon). Myelin removal was performed by adding 100 μl Myelin Removal Beads II (Miltenyi Biotec) per dorsal spinal cord and loaded in pre-wet LS columns (Miltenyi Biotec) on a MACS magnet stand. Cells in the flow-through were collected and washed for standard FACS staining.

### FACS of spinal microglia

To sort microglia using FACS, cells were incubated with anti-rat CD16/CD32 antibodies (1:100, BD Biosciences) for 15 min on ice. Before antibody staining, samples were incubated with the Zombie NIR™ Fixable Viability Kit (BioLegend) for dead cell exclusion during FACS. Then cells were incubated with covalently conjugated fluorochromes for 45 min on ice in a shaker. Finally, cells were resuspended in 300 μl FACS buffer, and 30 μl of Precision Count Beads™ (BioLegend) were added to quantify the number of cells expressing markers of interest. Samples were run using BD FACSAria™ II (BD Bioscience) and analysed using FlowJo software (v10.1; Tree Start Inc.). Cells were sorted directly into a 1.5-ml Eppendorf tube containing 350 μl RLT buffer CD45-BV785 (0.5 μg/ml, clone 30-F11, BioLegend), CD11b-BV421 (0.5 μg/ml, clone M1/70, BioLegend), CX3CR1-AF488 (1 μg/ml, clone SA011F11, BioLegend), P2Y12-PE (1 μg/ml, clone S16007D, BioLegend), TLR4-PECy7 (1 μg/ml, clone SA15-21, BioLegend), MHCII-PerCp/Cy5.5 (1 μg/ml, clone M5/114, BioLegend), CD11c-BV650 (2 μg/ml, clone N418, BioLegend), MerTK-BV605 (1 μg/ml, clone 2B10C42, BioLegend), CCR2-BV711 (0.5 μg/ml; Clone 475301, BD Bioscience), Ly6C-APC (1 μg/ml, clone HK1.4, Thermo Fisher Scientific). The number of live cells (Zombie NIR negative) in sorting experiments was 39,902 ± 7234.

### Bulk RNA-seq library preparation

For bulk RNA-seq, RNA was extracted from 2000 CD45^low^/CD11b^+^/CX3CR1^+^ cells per sample using RNeasy Micro Kit (QIAGEN 74004) and eluted with 14 μl nuclease-free water. Following this, RNA samples were examined using Agilent Bioanalyzer (Agilent Technologies, Palo Alto, CA, USA) for purity and quality. Total RNA stored in RNase-free water was amplified using SMART-Seq™ v4 Ultra™ Low Input RNA Kit for Sequencing (Takara Bio USA, Inc., Mountain View, USA) with the double strand cDNA (ds-cDNA) being synthesised. Then ds-cDNA was purified with AMPure XP beads and quantified with Qubit (Life Technologies™). For library preparation, 1 ng cDNA per sample was used as input to construct 250–300 bp insert cDNA libraries using NEBNext® UltraTM RNA Library Prep Kit (New England Biolabs, Ipswich, USA) following the manufacturer’s recommendations. Libraries were sequenced by Novogene as 150-bp paired-end reads on an Illumina Novaseq 6000 platform. Library preparation and sequencing were contacted on the premises of Novogene Europe (Cambridge, UK).

### RNA-seq data processing and gene expression analysis

All sequencing data processing was performed on a Unix-based operating system server at Eli Lilly. Raw files were demultiplexed into FASTQ files (Phred(Q) ≥ 35) and checked for potential contamination. The FASTQ files underwent quality control (QC) assessments using FastQC (v0.11.9). Sequences were aligned using HiSat2^83^ (version 2.1) to mm39 (GRCm39.105) reference mouse genome and using gene annotations release 104 obtained from Ensembl. Gene expression quantification (quantification of fragments or templates, hereby referred to as read counts) against the standard (spliced) reference genome was achieved using featureCounts^84^ (version 1.5.2). A quality check of aligned data was performed using Picard (http://broadinstitute.github.io/picard/), and results were visualised with MultiQC^85^.

All analyses were performed in R (version 4.2.0). Read counts were analysed for differential expression using the R package DESeq2^86^ (version 1.37.0) downloaded from Bioconductor. Datasets were filtered for non-expressed and lowly expressed genes (minimum of 10 counts in at least 50% of the samples). *p*-values were adjusted for multiple testing using the false discovery rate (FDR) method. Significant differential expression was inferred based on the co-occurrence of an absolute log_2_ fold change >1 and an FDR-adjusted *p*-value <0.05. Enrichment for GO terms for individual comparisons was performed using the EnrichGO function from the clusterProfiler R package in Bioconductor. A *p*-value and *q*-value cutoff of 0.05 was used. Ingenuity pathway analysis (IPA) (Ingenuity Systems, 01-20-04, http://www.ingenuity.com) was used to analyse upstream regulators.

### Gene set enrichment analysis (GSEA)

The K/BxN-specific transcriptional profile of DH microglia in WT was compared with microglia transcriptomes from mouse models of neuropathic pain such as microarray analysis of dorsal horn microglia in WT mice after spinal nerve L4 transection and L3 left intact^36^, RNA-seq analysis of dorsal horn microglia in WT mice with partial sciatic nerve ligation^35^ and RNA-seq analysis of microglia in the ipsilateral lumbar spinal cord after chronic constriction injury^34^ (GSE60670, GSE71133, GSE180627), ALS (SOD1) (GSE43366), AD (5xFAD) (GSE98969), thalamus in RA (TNFtg) (GSE145708), LPS (GSE115571). All signatures were derived from publicly available transcriptomes downloaded from Gene Expression Omnibus (GEO). The statistical significance of GSEA results was assessed using 1000 sample permutations. A nominal *p*-value less than 0.001 was used to determine pairwise transcriptome connectivity. The normalised enrichment score (NES) accounts for differences in gene set size and in statistically significant and concordant correlations between GEO gene sets and the K/BxN dataset^87^. The accession number for the raw sequencing data reported in this paper is GEO: GSE213157.

### Isolation and functional studies in synaptosomes

Synaptosomes were isolated from the dorsal lumbar spinal cord of WT Control and K/BxN ST using a synaptic protein extraction reagent (Syn-PER; ThermoFisher Scientific). The resulting synaptosomes were collected and incubated with capsaicin or saline for 3 h in HBSS solution containing 1 mM Calcium. Gal-3 levels were quantified with western immunoblotting. Briefly, samples were run on a 10% w/v sodium dodecyl sulfate polyacrylamide tris-glycine gel (BioRad Cat# 4561035), transferred on PVDF membranes, and analysed by immunoblotting using Gal-3 (1:300, AF1197, R&D System) and GAPDH (1:5000, ab8245, Abcam).

### Statistics

All data are presented as means ± SEM, where *n* is the number of biological replicates (individual mice), with differences between means considered statistically significant when *p* < 0.05. All statistical analyses for behavioural and immunohistochemical data were conducted using GraphPad Prism (v8.3.0; GraphPad Software, USA). For any single comparison between two groups, an unpaired Student’s *t*-test (two groups using different samples) was used. Multiple comparison of data used one-way ANOVA followed by posthoc Tukey test if more than two groups are present or two-way repeated measure ANOVA followed by posthoc Tukey test for statistical analysis of behavioural testing, clinical scoring and western immunoblotting.

### Reporting summary

Further information on research design is available in the Nature Portfolio Reporting Summary linked to this article.

## Supplementary information

Supplementary Information

Supplementary Dataset 1

Supplementary Dataset 2

Supplementary Dataset 3

Reporting Summary

## Data Availability

The data that support the findings of this study are provided in the Source data file. RNA-sequencing data generated in this study have been deposited in the GEO database under the accession code GSE213157. Datasets used in GSEA analysis are deposited in the GEO database under: GSE60670, GSE71133, GSE180627, GSE43366, GSE98969, GSE145708, GSE115571. Raw data from every experiment have been deposited at https://figshare.com/s/d50817b964caf75c3f26. Source data are provided with this paper.

## Source data

Source Data

## Code availability

The code used throughout this study is available on GitHub.
