# Supplementary material for: Galectin-3 activates spinal microglia to induce inflammatory nociception in wild type but not in mice modelling Alzheimer’s disease
Source: Nat Commun. 2023 Jun 22;14:3579. doi: 10.1038/s41467-023-39077-1 (PMC10287730; doi:10.1038/s41467-023-39077-1)
Supplement: Supplementary file 1 — Supplementary Information [file 41467_2023_39077_MOESM1_ESM.pdf]

Supplementary Information

Galectin-3 activates spinal microglia to induce inflammatory nociception in wild type but not in mice modelling Alzheimer's Disease

Sideris-Lampretsas et al.

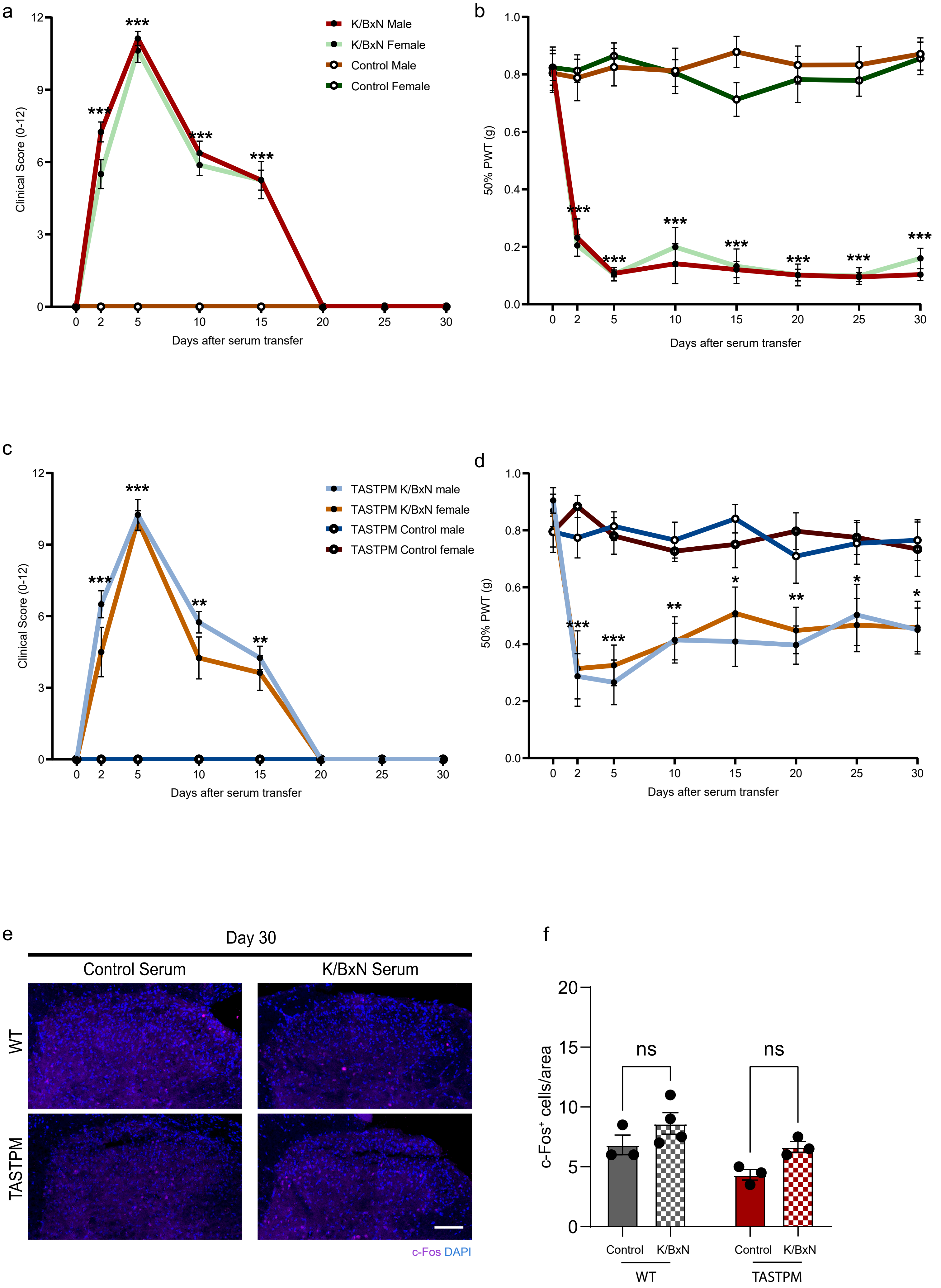
a
Clinical Score (0-12)
Days after serum transfer
K/BxN Male
K/BxN Female
Control Male
Control Female
***
b
50% PWT (g)
Days after serum transfer
***
c
Clinical Score (0-12)
Days after serum transfer
TASTPM K/BxN male
TASTPM K/BxN female
TASTPM Control male
TASTPM Control female
***
**
d
50% PWT (g)
Days after serum transfer
***
**
*
e
Day 30
Control Serum
K/BxN Serum
WT
TASTPM
c-Fos DAPI
f
c-Fos⁺ cells/area
ns
Control
K/BxN
WT
TASTPM

Supplementary figures

Supplementary Figure 1: No sex-dependent changes in clinical scores and mechanical hypersensitivity in WT and TASTPM K/BxN ST. (a) Clinical scores of fore and hind paws and (b) mechanical hypersensitivity in male and female WT after K/BxN ST. (c) Clinical scores of fore and hind paws and (d) mechanical hypersensitivity in male and female TASTPM after K/BxN ST. n = 8 male and female mice per group. Data represent mean ± SEM. ns – not significant, *$p < 0.05$, **$p < 0.01$, ***$p < 0.001$, Two-Way RM ANOVA, Tukey's multiple comparisons test. (e) Representative images of c-Fos$^+$ neurons in DH. Scalebar, 100μm (f) Quantification of c-Fos$^+$ neurons in WT and TASTPM K/BxN ST at Day 30. n = 3 (WT and TASTPM Control ST and TASTPM K/BxN ST), n = 4 (WT K/BxN ST). Data represent mean ± SEM. Two-way ANOVA, Tukey's multiple comparisons test.

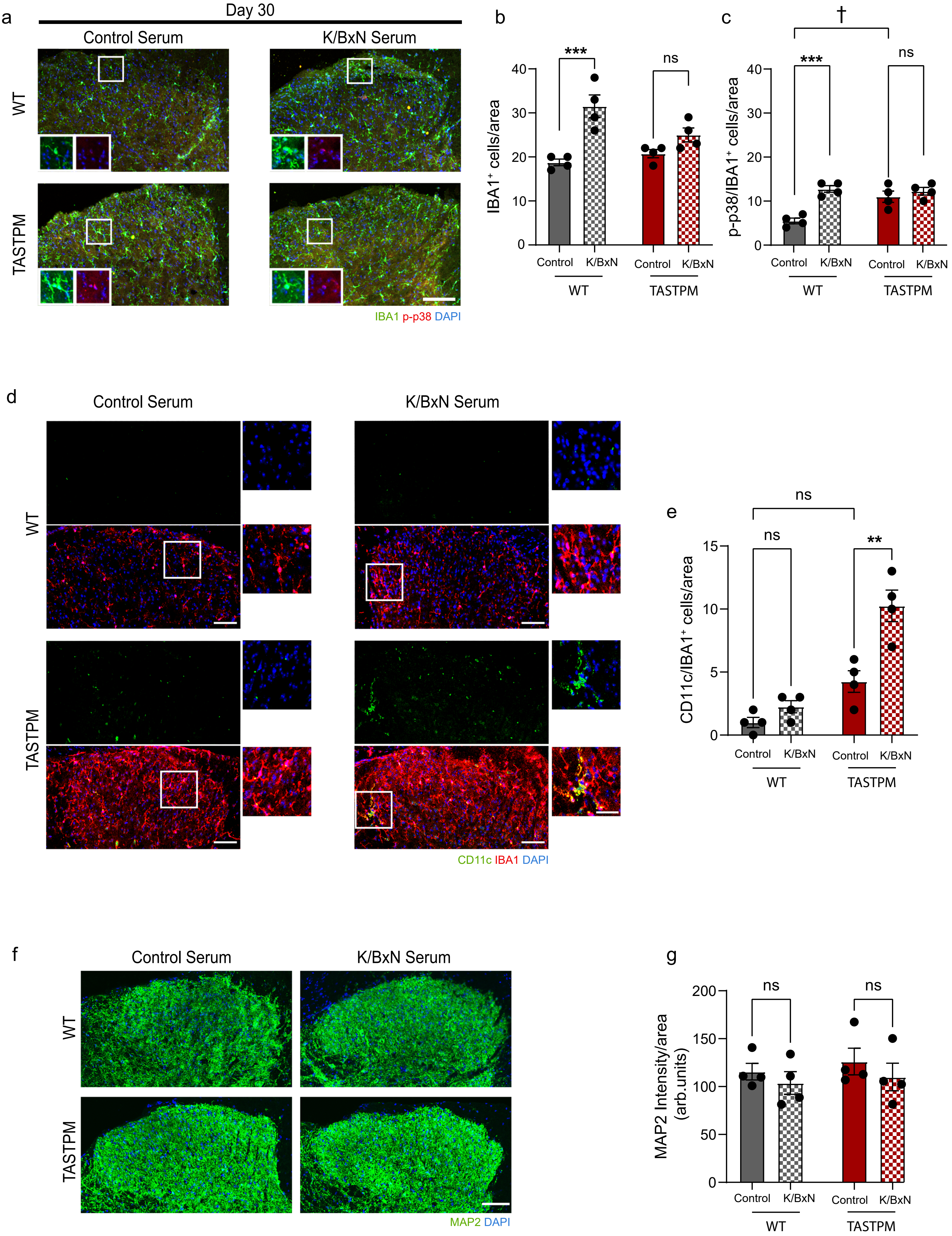
a
Day 30
Control Serum
K/BxN Serum
WT
TASTPM
IBA1 p-p38 DAPI
b
***
ns
IBA1⁺ cells/area
40
30
20
10
0
Control
K/BxN
Control
K/BxN
WT
TASTPM
c
†
***
ns
p-p38/IBA1⁺ cells/area
40
30
20
10
0
Control
K/BxN
Control
K/BxN
WT
TASTPM
d
Control Serum
K/BxN Serum
WT
TASTPM
CD11c IBA1 DAPI
e
ns
ns
**
CD11c/IBA1⁺ cells/area
15
10
5
0
Control
K/BxN
Control
K/BxN
WT
TASTPM
f
Control Serum
K/BxN Serum
WT
TASTPM
MAP2 DAPI
g
ns
ns
MAP2 Intensity/area (arb.units)
200
150
100
50
0
Control
K/BxN
Control
K/BxN
WT
TASTPM

Supplementary Figure 2: Changes in microglial markers in WT and TASTPM after K/BxN ST. (a) Increased number of $pp38^+$ microglia in the dorsal horn of WT and TASTPM K/BxN ST at day 30. Scalebar, 100µm (b, c) Quantification of $IBA1^+$ and p-p38/$IBA1^+$ microglia in DH at Day 30. Data represent mean ± SEM, n = 4 mice per group. ns – not significant, ***$p < 0.001$, ****$p < 0.0001$, †$p < 0.05$. Two-way ANOVA, Tukey's multiple comparisons test. (d) Increased number of $CD11c^+$ microglia in DH of TASTPM at day 5 K/BxN ST. Scalebar, 100 µm (e) Quantification of CD11c/$IBA1^+$ microglia. Data represent mean ± SEM, n = 4 mice per group. **$p = 0.0011$. Two-way ANOVA, Tukey's multiple comparisons test. (f) No change in MAP2 intensity in DH of WT and TASTPM at day 5 K/BxN ST. Scalebar, 100 µm (e) Quantification of MAP2 intensity. Data represent mean ± SEM, n = 4 mice per group. ns – not significant, Two-way ANOVA, Tukey's multiple comparisons test.

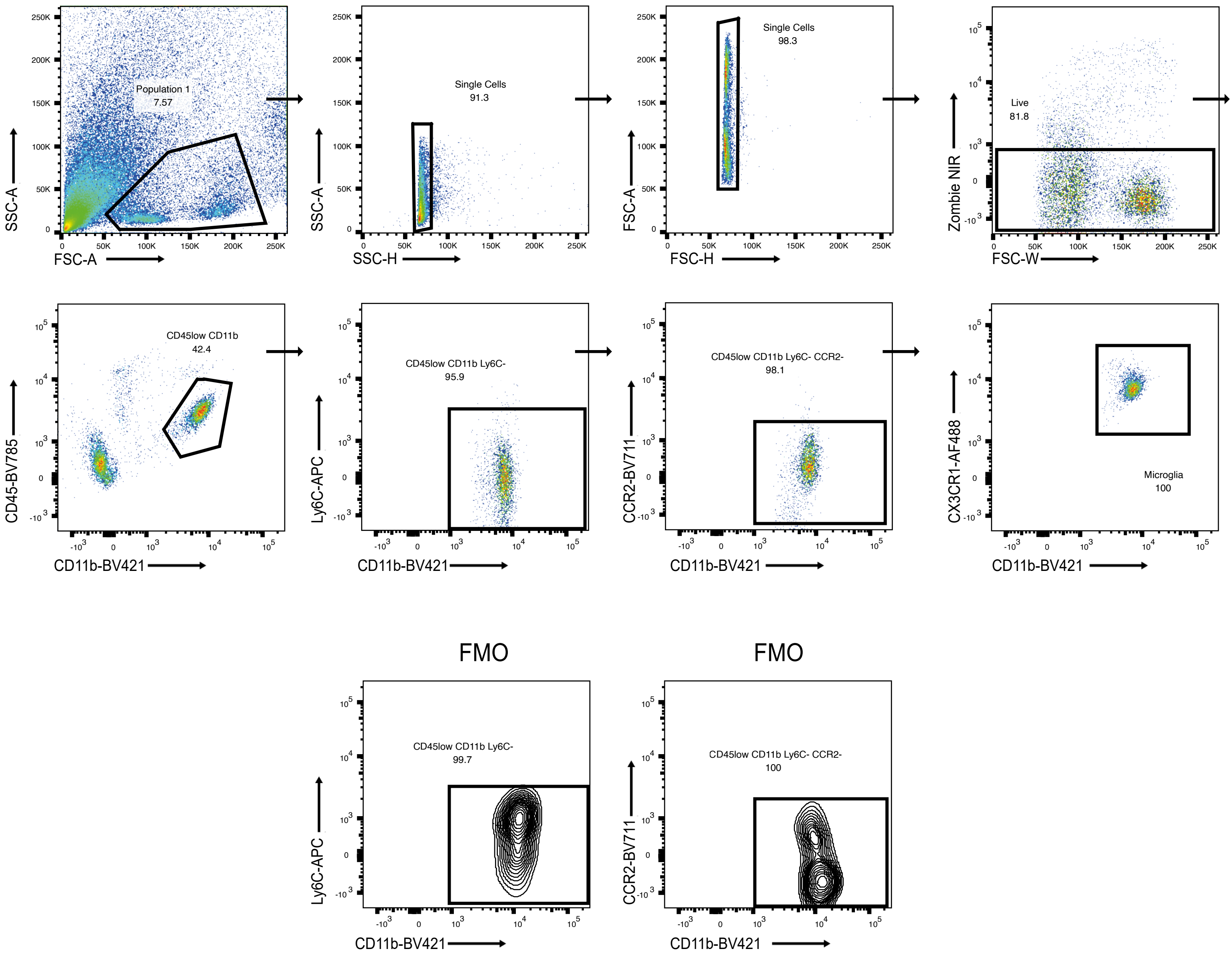

Population 1
7.57
SSC-A
FSC-A
Single Cells
91.3
SSC-A
SSC-H
Single Cells
98.3
FSC-A
FSC-H
Live
81.8
Zombie NIR
FSC-W
CD45low CD11b
42.4
CD45-BV785
CD11b-BV421
CD45low CD11b Ly6C-
95.9
Ly6C-APC
CD11b-BV421
CD45low CD11b Ly6C- CCR2-
98.1
CCR2-BV711
CD11b-BV421
Microglia
100
CX3CR1-AF488
CD11b-BV421
FMO
CD45low CD11b Ly6C-
99.7
Ly6C-APC
CD11b-BV421
FMO
CD45low CD11b Ly6C- CCR2-
100
CCR2-BV711
CD11b-BV421

Supplementary Figure 3: Gating Strategy for microglia isolation using FACS. $CD45^{low}$ $CD11b^{+}$ $Ly6C^{-}$ $CCR2^{-}$ $CX3CR1^{+}$ cells were isolated per dorsal spinal cord.

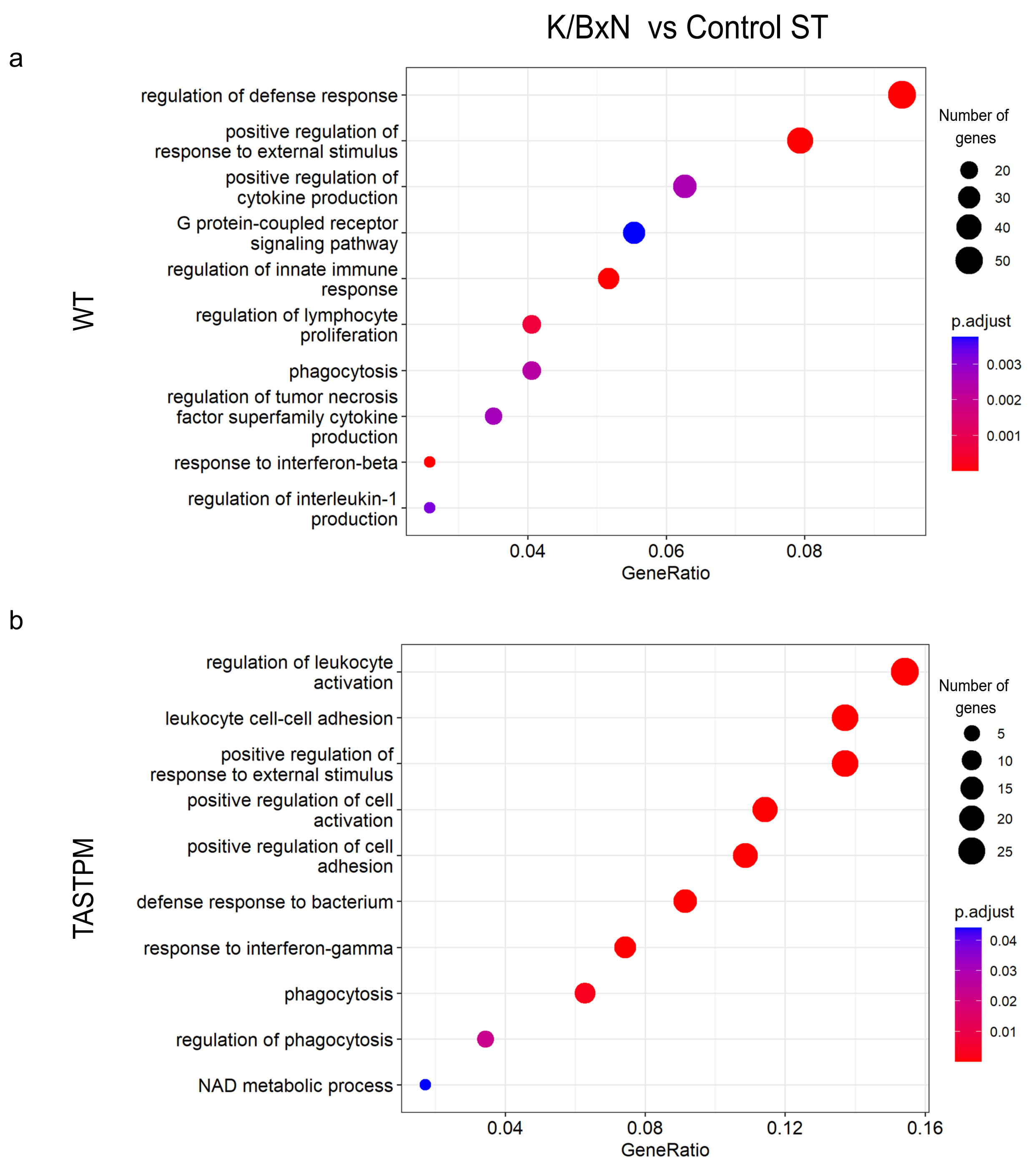

K/BxN vs Control ST
a
WT
regulation of defense response
positive regulation of response to external stimulus
positive regulation of cytokine production
G protein-coupled receptor signaling pathway
regulation of innate immune response
regulation of lymphocyte proliferation
phagocytosis
regulation of tumor necrosis factor superfamily cytokine production
response to interferon-beta
regulation of interleukin-1 production
0.04
0.06
0.08
GeneRatio
Number of genes
20
30
40
50
p.adjust
0.003
0.002
0.001
b
TASTPM
regulation of leukocyte activation
leukocyte cell-cell adhesion
positive regulation of response to external stimulus
positive regulation of cell activation
positive regulation of cell adhesion
defense response to bacterium
response to interferon-gamma
phagocytosis
regulation of phagocytosis
NAD metabolic process
0.04
0.08
0.12
0.16
GeneRatio
Number of genes
5
10
15
20
25
p.adjust
0.04
0.03
0.02
0.01

Supplementary Figure 4: Transcripts expressed by dorsal horn microglia in the WT K/BxN ST are enriched in pathways associated with IL-1β, TNF-α and IFN-β signalling, while microglia in the TASTPM K/BxN ST show upregulation of transcripts involved in glycolysis and metabolism. (a) Top 10 Gene Ontology (GO) pathways overrepresented in DEGs in DH microglia in the WT K/BxN ST. (b) Top 10 Gene Ontology (GO) pathways overrepresented in DEGs in DH microglia in the TASTPM K/BxN ST. GeneRatio is the number of observed genes divided by the number of expected genes from each GO. n = 3 (WT and TASTPM Control), n = 4 (WT and TASTPM K/BxN ST).

a

b

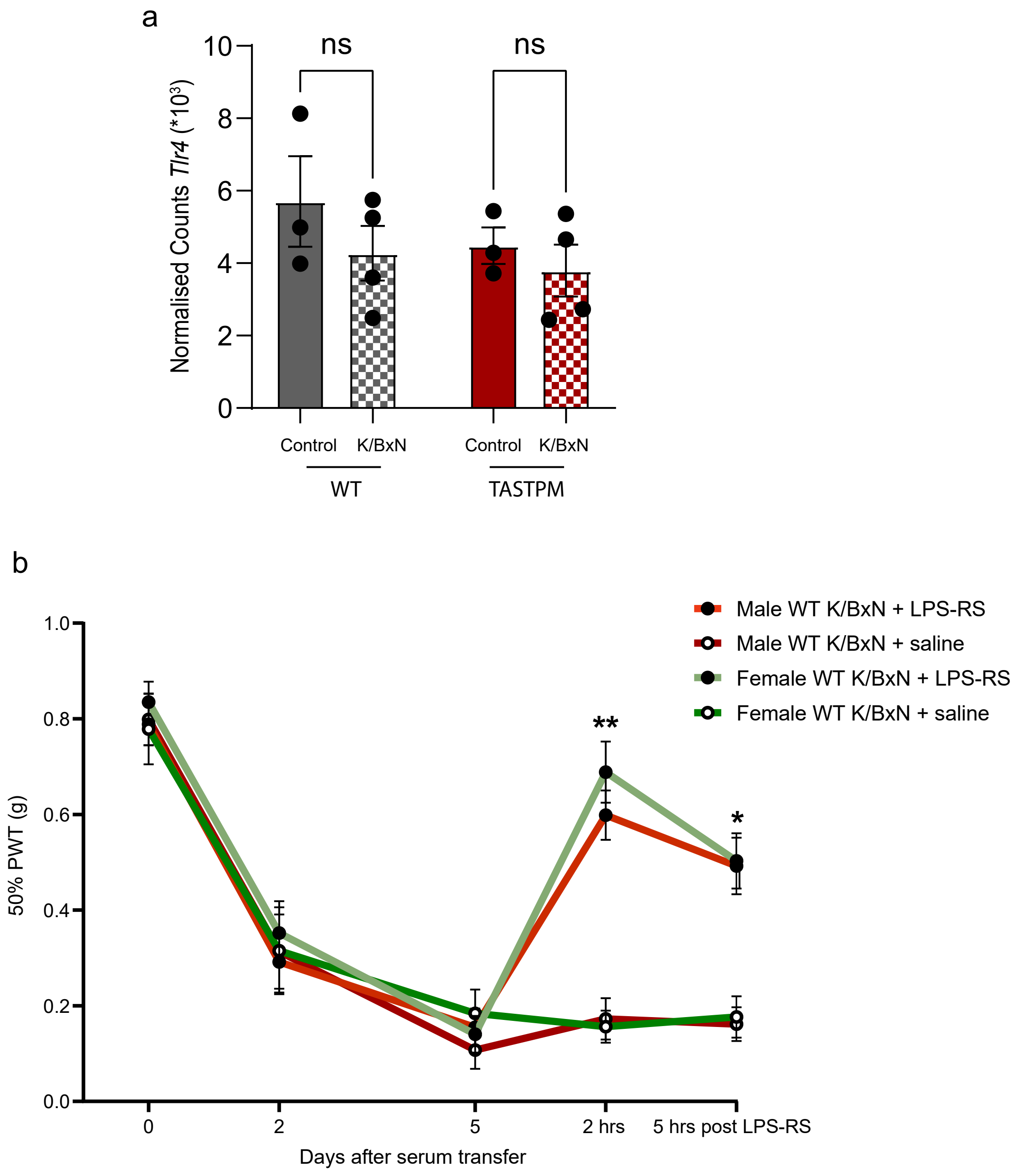

ns
ns
Normalised Counts Tlr4 (*10³)
10
8
6
4
2
0
Control
K/BxN
Control
K/BxN
WT
TASTPM
Male WT K/BxN + LPS-RS
Male WT K/BxN + saline
Female WT K/BxN + LPS-RS
Female WT K/BxN + saline
50% PWT (g)
1.0
0.8
0.6
0.4
0.2
0.0
**
*
0
2
5
2 hrs
5 hrs post LPS-RS
Days after serum transfer

Supplementary Figure 5: Spinal TLR4 in K/BxN ST-induced inflammatory allodynia. (a) Quantification of normalised counts from RNA-seq for *Tlr4*. Normalised counts were calculated using variance stabilizing transformation from R package DESeq2. ns – not significant. n = 3 (WT and TASTPM Control), n = 4 (WT and TASTPM K/BxN ST). (b) Intrathecal injection of a TLR4 antagonist (LPS-RS) (10 μg/ 5 μl/mouse) reversed mechanical hypersensitivity both in male and female WT K/BxN ST. Data represent mean ± SEM, n = 8 mice per group. *p < 0.05, **p < 0.01, compared to saline-treated same sex WT K/BxN ST, Two-Way RM ANOVA, Tukey's multiple comparisons test.

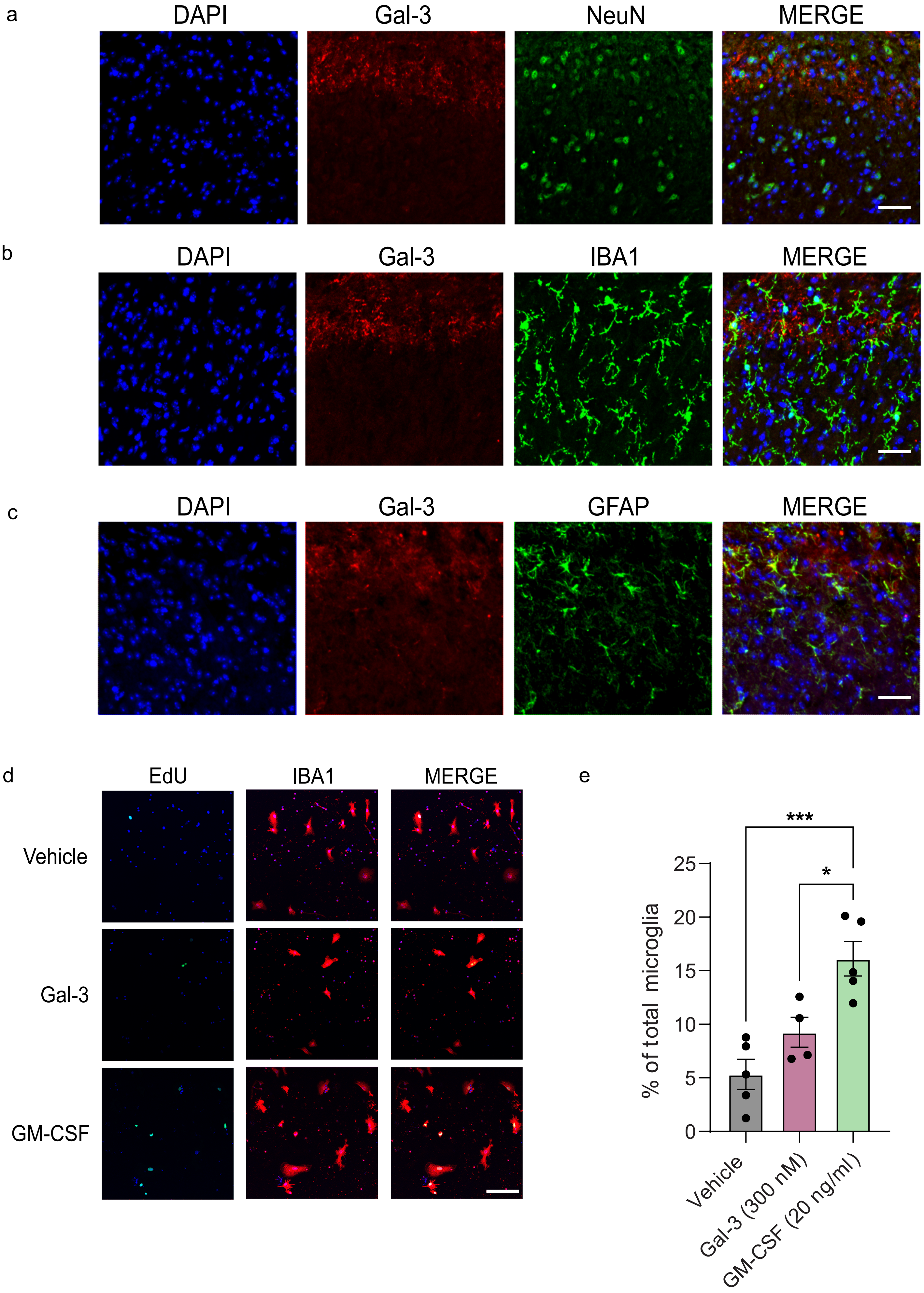
a
DAPI
Gal-3
NeuN
MERGE
b
DAPI
Gal-3
IBA1
MERGE
c
DAPI
Gal-3
GFAP
MERGE
d
EdU
IBA1
MERGE
Vehicle
Gal-3
GM-CSF
e
***
*
% of total microglia
0
5
10
15
20
25
Vehicle
Gal-3 (300 nM)
GM-CSF (20 ng/ml)

Supplementary Figure 6: Gal-3 is minimally expressed by microglia and astrocytes in WT mice, and not expressed by dorsal horn projection neurons. (a) Representative images of Gal-3 and NeuN in the dorsal lumbar spinal cord. (b) Representative images of Gal-3 and IBA1 in DH. (c) Representative images of Gal-3 and GFAP in DH. Scalebar, 20 μm. (d) Gal-3 (300 nM) did not induce proliferation in in vitro cultured primary microglia. (e) Quantification of percentage of EdU/IBA1^{+} microglia. Data represent mean ± SEM, n = 4 (Gal-3), n = 5 (GM-CSF was used as a positive control). *p = 0.0264, ***p = 0.0008. One-way ANOVA, Bonferroni's multiple comparisons test.

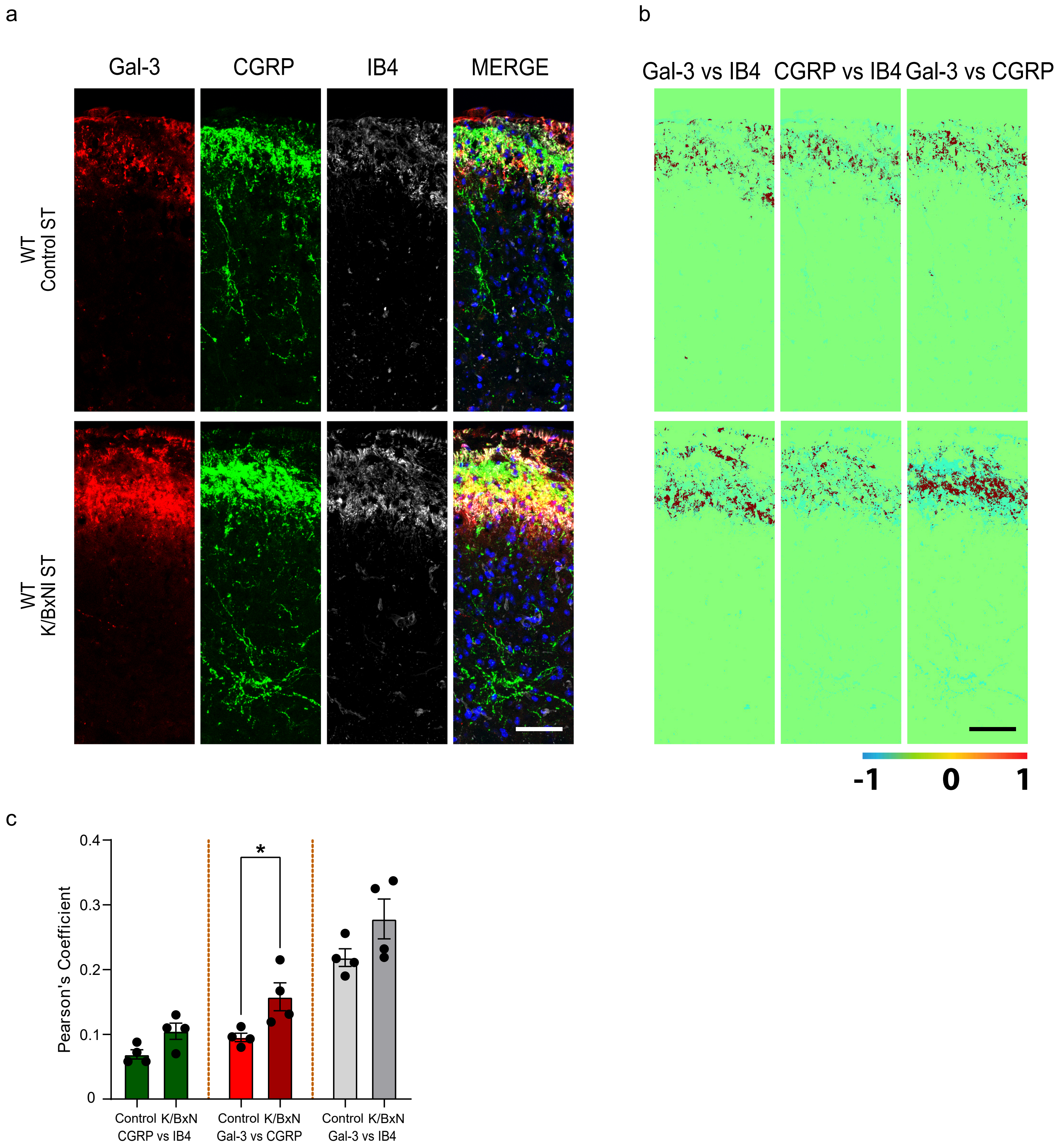

a
Gal-3
CGRP
IB4
MERGE
WT
Control ST
WT
K/BxNI ST
b
Gal-3 vs IB4
CGRP vs IB4
Gal-3 vs CGRP
-1
0
1
c
Pearson's Coefficient
0.4
0.3
0.2
0.1
0
*
Control
K/BxN
CGRP vs IB4
Control
K/BxN
Gal-3 vs CGRP
Control
K/BxN
Gal-3 vs IB4

Supplementary Figure 7: Gal-3 is normally expressed in non-peptidergic IB4$^+$ terminals in DH superficial laminae, but also in peptidergic CGRP terminals in K/BxN ST at day 5. (a) Representative images of immunostaining in primary afferent nerve terminals of control and K/BxN ST DH. Scalebar, 20µm, (b) Representative colocalization heatmaps generated by Colocalization Colormap. (c) Quantification of colocalising pixel proportion shows more Gal-3$^+$ terminals colocalised with CGRP$^+$ terminals in the superficial laminae of the dorsal lumbar spinal cord in WT K/BxN ST. Data represent mean ± SEM. n = 4 mice per group. *p = 0.0319, Unpaired Student's *t*-test, two-tailed.

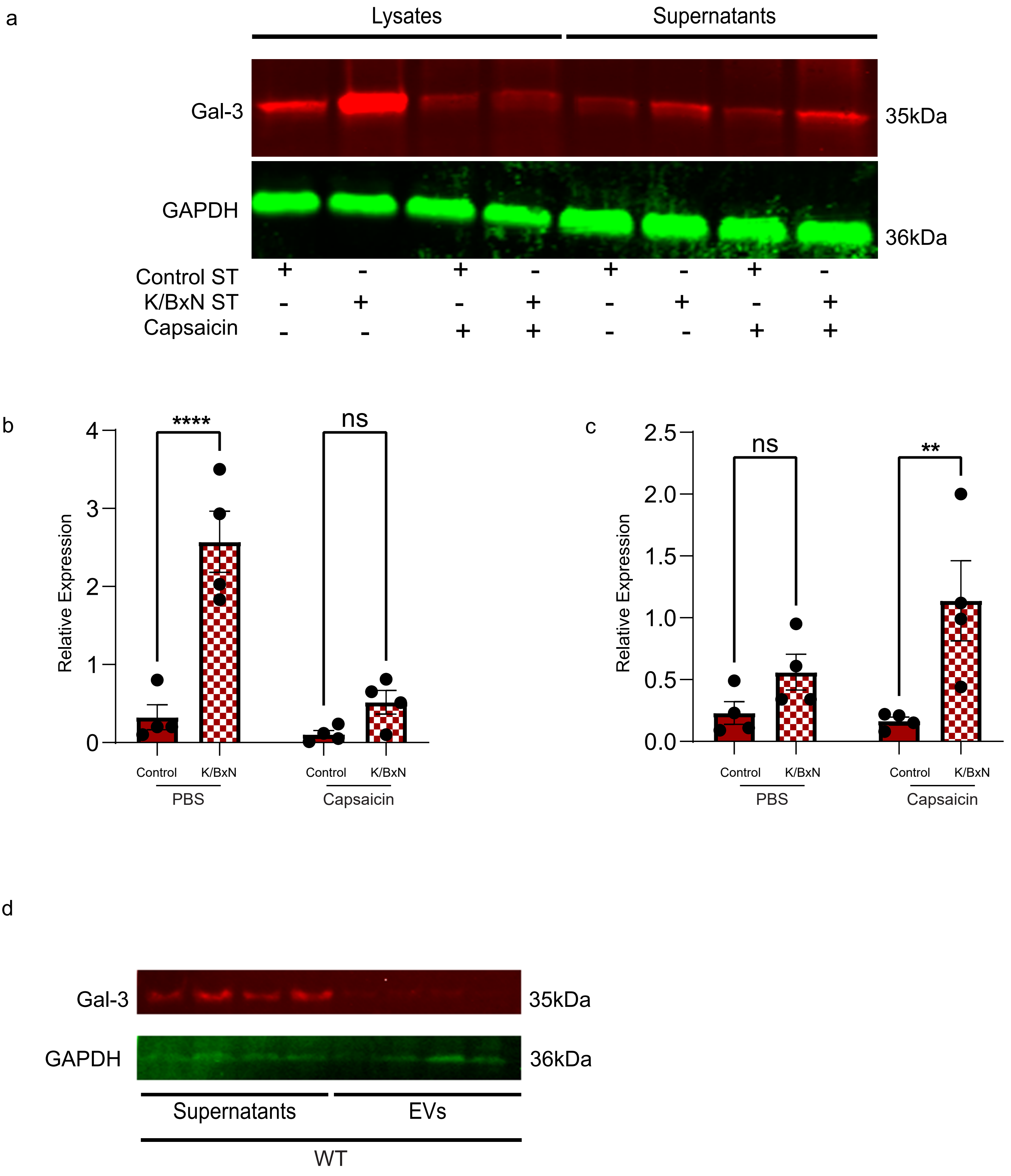
a
Lysates
Supernatants
Gal-3
35kDa
GAPDH
36kDa
Control ST + - + - + - + -
K/BxN ST - + - + - + - +
Capsaicin - - + + - - + +
b
Relative Expression
****
ns
Control
K/BxN
PBS
Capsaicin
c
Relative Expression
ns
**
Control
K/BxN
PBS
Capsaicin
d
Gal-3
35kDa
GAPDH
36kDa
Supernatants
EVs
WT

Supplementary Figure 8: Gal-3 is released by capsaicin in TASTPM control and K/BxN ST DH synaptosomes. (a) Western blots and quantification of Gal-3 in synaptosome lysates (b) and supernatants (c) after incubation of capsaicin (1 μM for 3 h). Data represent mean ± SEM, n = 4 mice per group. ns – not significant, $^{**}p = 0.0056$, $^{****}p < 0.0001$, Two-way ANOVA, Tukey's multiple comparisons test. (d) Representative Western blots showing no Gal-3 detection in the EV fraction of synaptosome preparations from WT K/BxN ST.

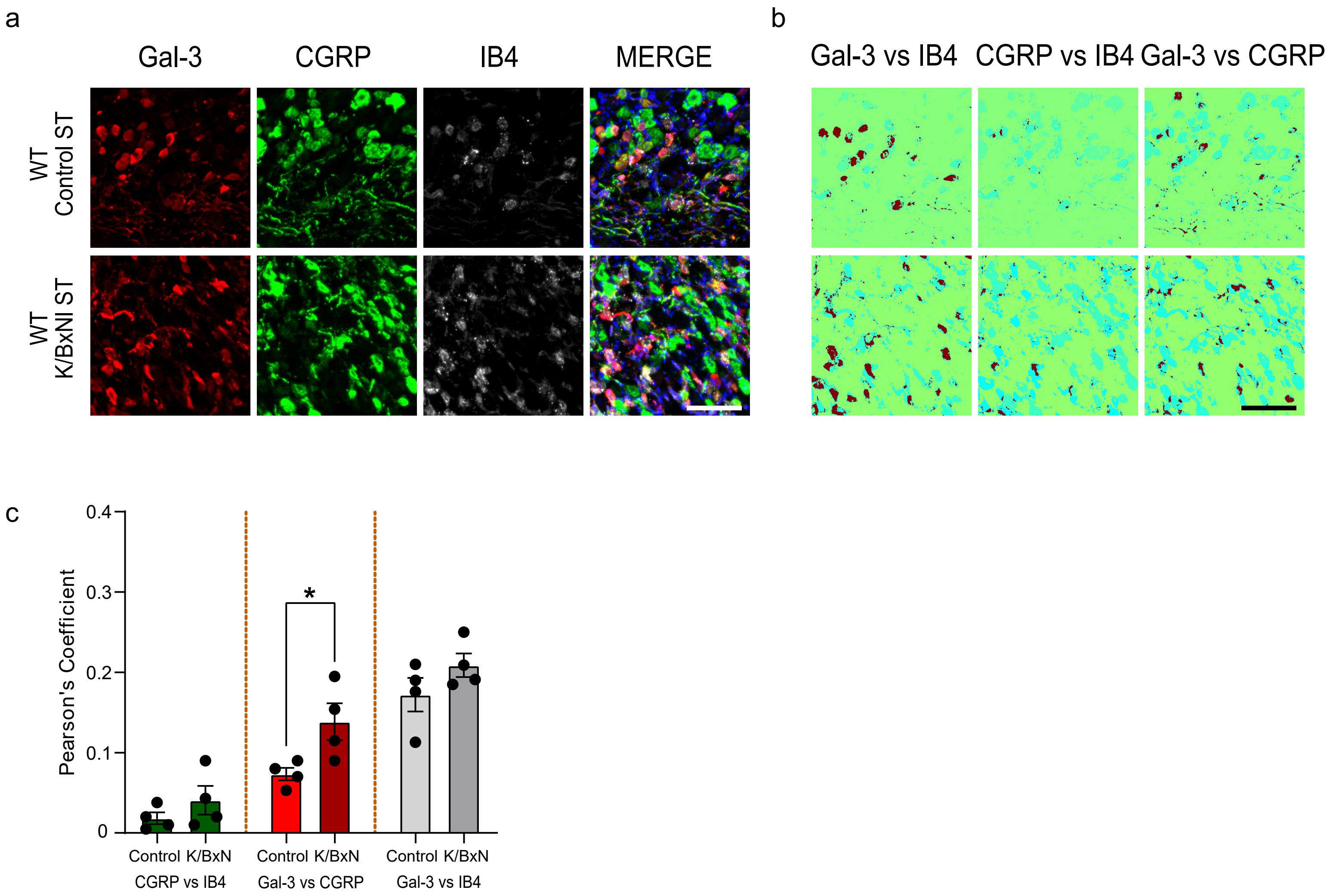
a
Gal-3
CGRP
IB4
MERGE
WT Control ST
WT K/BxNI ST
b
Gal-3 vs IB4
CGRP vs IB4
Gal-3 vs CGRP
c
Pearson's Coefficient
0.4
0.3
0.2
0.1
0
*
Control
K/BxN
CGRP vs IB4
Gal-3 vs CGRP
Gal-3 vs IB4

Supplementary Figure 9: In the dorsal root ganglia, Gal-3 is normally expressed in non-peptidergic IB4$^+$ neurons, and expression is also evident in peptidergic CGRP neurons in K/BxN ST at day 5. (a) Representative images of control and K/BxN ST lumbar DRGs. Scalebar, 20 μm, (b) Representative colocalization heatmaps generated by Colocalization Colormap. (c) Quantification of colocalising pixel proportions shows that more Gal-3$^+$ neurons colocalised with CGRP$^+$ neurons in day 5 K/BxN ST than control ST DRGs. Data represent mean ± SEM, n = 4 mice per group, *p = 0.0363, Unpaired Student's *t*-test, two-tailed.

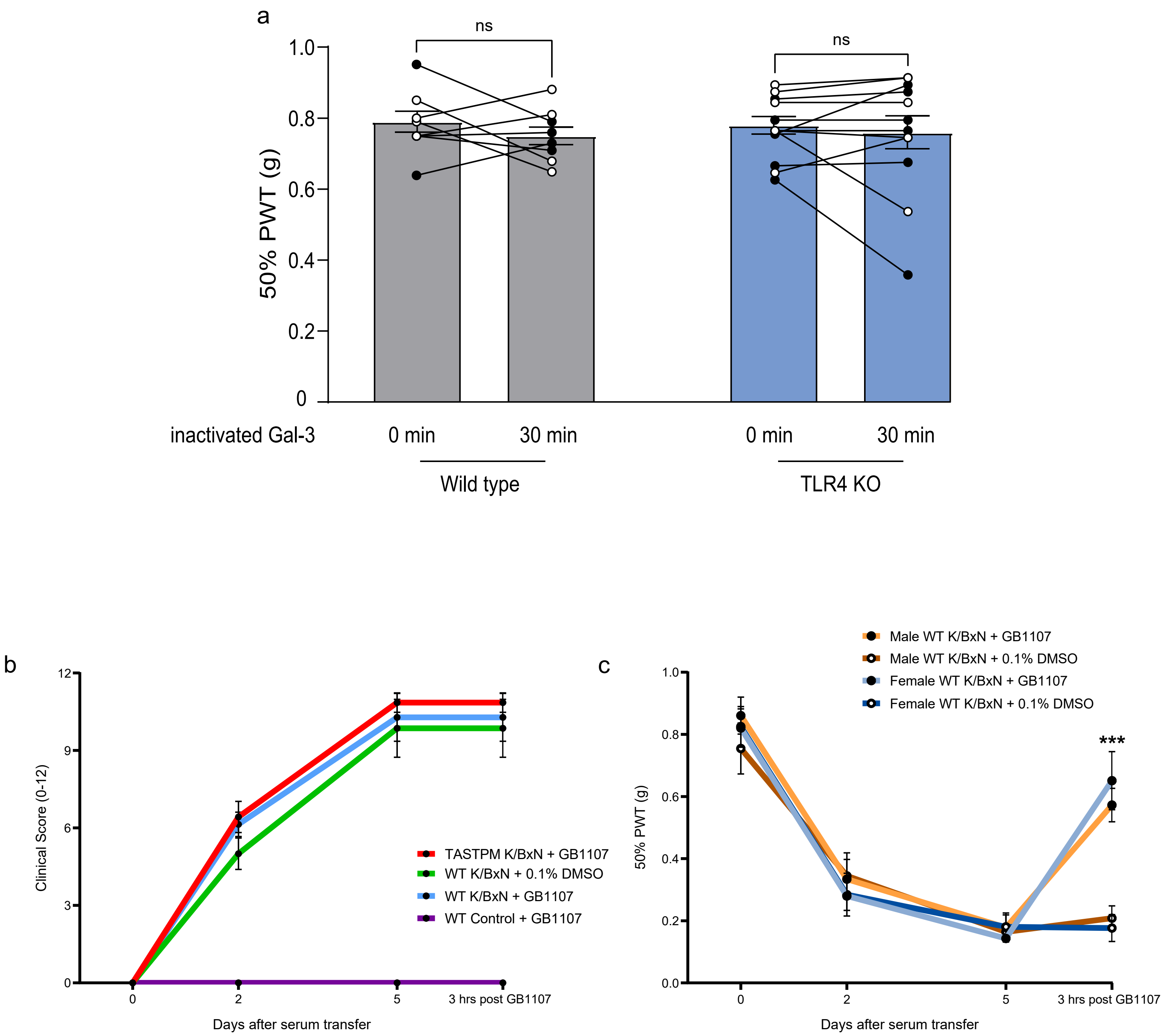

a
ns
ns
1.0
0.8
0.6
0.4
0.2
0
50% PWT (g)
inactivated Gal-3
0 min
30 min
0 min
30 min
Wild type
TLR4 KO
b
12
9
6
3
0
Clinical Score (0-12)
TASTPM K/BxN + GB1107
WT K/BxN + 0.1% DMSO
WT K/BxN + GB1107
WT Control + GB1107
0
2
5
3 hrs post GB1107
Days after serum transfer
c
Male WT K/BxN + GB1107
Male WT K/BxN + 0.1% DMSO
Female WT K/BxN + GB1107
Female WT K/BxN + 0.1% DMSO
1.0
0.8
0.6
0.4
0.2
0.0
50% PWT (g)
***
0
2
5
3 hrs post GB1107
Days after serum transfer

Supplementary Figure 10: Intrathecal injection of heat-inactivated Gal-3 shows no effect on WT and TLR4 KO mechanical thresholds. (a) Mechanical thresholds were assessed before and 30 min after intrathecal injections of heat-inactivated Gal-3 (1 μM). Data represent mean ± SEM, n = 7 (WT Control ST), n = 8 (WT K/BxN ST), and n = 4 (TASTPM Control and K/BxN ST). Two-Way RM ANOVA, Tukey's multiple comparisons test. (b) clinical scoring in both WT and TASTPM K/BxN ST was not affected by GB1107 (10 nM intrathecal), Two-Way RM ANOVA, Tukey's multiple comparisons test. (c) GB1107 (10 nM) reversed mechanical hypersensitivity both in male and female WT K/BxN ST mice. Data represent mean ± SEM, n = 8 mice per group. ***$p < 0.001$ GB1107-treated vs DMSO-treated same sex WT K/BxN ST, Two-Way RM ANOVA, Tukey's multiple comparisons test.
