# Supplementary material for: Galectin-3 activates spinal microglia to induce inflammatory nociception in wild type but not in mice modelling Alzheimer’s disease
Source: Nat Commun. 2023 Jun 22;14:3579. doi: 10.1038/s41467-023-39077-1 (PMC10287730; doi:10.1038/s41467-023-39077-1)
Supplement: Supplementary file 5 — Reporting Summary [file 41467_2023_39077_MOESM5_ESM.pdf]

nature portfolio

Corresponding author(s): Marzia Malcangio

Last updated by author(s): Apr 19, 2023

Reporting Summary

Nature Portfolio wishes to improve the reproducibility of the work that we publish. This form provides structure for consistency and transparency in reporting. For further information on Nature Portfolio policies, see our Editorial Policies and the Editorial Policy Checklist.

Statistics

For all statistical analyses, confirm that the following items are present in the figure legend, table legend, main text, or Methods section.

n/a	Confirmed	
[ ]	[x]	The exact sample size (*n*) for each experimental group/condition, given as a discrete number and unit of measurement
[ ]	[x]	A statement on whether measurements were taken from distinct samples or whether the same sample was measured repeatedly
[ ]	[x]	The statistical test(s) used AND whether they are one- or two-sided<br>*Only common tests should be described solely by name; describe more complex techniques in the Methods section.*
[ ]	[x]	A description of all covariates tested
[ ]	[x]	A description of any assumptions or corrections, such as tests of normality and adjustment for multiple comparisons
[ ]	[x]	A full description of the statistical parameters including central tendency (e.g. means) or other basic estimates (e.g. regression coefficient) AND variation (e.g. standard deviation) or associated estimates of uncertainty (e.g. confidence intervals)
[ ]	[x]	For null hypothesis testing, the test statistic (e.g. *F*, *t*, *r*) with confidence intervals, effect sizes, degrees of freedom and *P* value noted<br>*Give P values as exact values whenever suitable.*
[x]	[ ]	For Bayesian analysis, information on the choice of priors and Markov chain Monte Carlo settings
[x]	[ ]	For hierarchical and complex designs, identification of the appropriate level for tests and full reporting of outcomes
[x]	[ ]	Estimates of effect sizes (e.g. Cohen's *d*, Pearson's *r*), indicating how they were calculated

Our web collection on statistics for biologists contains articles on many of the points above.

Software and code

Policy information about availability of computer code

Data collection	The following softwares were used to collect the data in this study:<br>- BD FACSDIVA (v.8.0.1)<br>- Zeiss Zen Black v.2011
Data analysis	The following softwares were used to analyze the data in this study:<br>- ImageJ (1.50i, Wayne Rasband, National Institutes of Health, USA)<br>- Prism v8.3.0 (GraphPad Software, Inc)<br>- FastQC v0.11.9<br>- HiSat2 (version 2.1)<br>- featureCounts v1.5.2)<br>- R v4.2.0<br>- DESeq2 v1.37.0<br>- clusterProfiler v4.4.4<br>- Ingenuity pathway analysis (01-20-04)<br>- FlowJo software v10.1 (Tree Star, Inc.)<br>- Custom code used for bulk RNA-seq analysis is available on Github.

For manuscripts utilizing custom algorithms or software that are central to the research but not yet described in published literature, software must be made available to editors and reviewers. We strongly encourage code deposition in a community repository (e.g. GitHub). See the Nature Portfolio guidelines for submitting code & software for further information.

Data

Policy information about availability of data

All manuscripts must include a data availability statement. This statement should provide the following information, where applicable:

- Accession codes, unique identifiers, or web links for publicly available datasets
- A description of any restrictions on data availability
- For clinical datasets or third party data, please ensure that the statement adheres to our policy

The data and code that support the findings of this study are available from the corresponding author upon request. The accession number for the raw sequencing data reported in this paper is GEO: GSE213157. Data for GSEA were accessed though GEO using: GSE60670, GSE180627, GSE71133, GSE43366, GSE98969, GSE145708, GSE115571. Custom code is available on Github (https://github.com/Sideris-George/Sideris-Lampretsas-et-al_2023_Nat_Coms).

Human research participants

Policy information about studies involving human research participants and Sex and Gender in Research.

Reporting on sex and gender	N/A
Population characteristics	N/A
Recruitment	N/A
Ethics oversight	N/A

Note that full information on the approval of the study protocol must also be provided in the manuscript.

Field-specific reporting

Please select the one below that is the best fit for your research. If you are not sure, read the appropriate sections before making your selection.

[x] Life sciences   [ ] Behavioural & social sciences   [ ] Ecological, evolutionary & environmental sciences

For a reference copy of the document with all sections, see nature.com/documents/nr-reporting-summary-flat.pdf

Life sciences study design

All studies must disclose on these points even when the disclosure is negative.

Sample size	Sample sizes were chosen to maximize certainty in experimental results, while also taking into consideration the ethical concerns of using mouse tissue. Sample sizes for behavioural experiments were chosen on the basis of standard power calculations (with $\alpha = 0.05$ and power of 0.8) performed for similar experiments that were previously published. A minimum of four biological replicates were included per experiment and within each, multiple cells across several slices were measured. These sample sizes were sufficient to detect the presence of effects or their absence, given the groups standard deviation and the sensitivity of measurements.
Data exclusions	No data points were excluded during the acquisition or analysis.
Replication	All experiments were repeated multiple times (at a minimum of three) to ensure the observed result could be replicated.<br>For behavioural experiments, 8-12 mice per group were used and each experiment was repeated three times with different blinded experimenters. The results were consistent in all cases where multiple researchers performed the same measurements.<br>The RNA-sequencing study was repeated once in four groups of 3-4 mice per group.
Randomization	Samples and mice were randomly allocated into treatment groups.
Blinding	Experimenters were blinded to treatment during behavioural testing and clinical scoring. Similarly, during data processing, experimenters were blinded to treatment and genotype.

Reporting for specific materials, systems and methods

We require information from authors about some types of materials, experimental systems and methods used in many studies. Here, indicate whether each material, system or method listed is relevant to your study. If you are not sure if a list item applies to your research, read the appropriate section before selecting a response.

Materials & experimental systems

n/a	Involved in the study
[ ]	[x] Antibodies
[x]	[ ] Eukaryotic cell lines
[x]	[ ] Palaeontology and archaeology
[ ]	[x] Animals and other organisms
[x]	[ ] Clinical data
[x]	[ ] Dual use research of concern

Methods

n/a	Involved in the study
[x]	[ ] ChIP-seq
[ ]	[x] Flow cytometry
[x]	[ ] MRI-based neuroimaging

Antibodies

Antibodies used	The following primary antibodies were used for IHC:<br>c-Fos (1:200, #2250, Cell Signaling), IBA1 (1:1000, Wako Chemicals; 1:300, ab5076, Abcam, Cambridge, United Kingdom), p-p38 (1:100, #9211, Cell Signaling), CD68 (1:200, ab125212, Abcam, Cambridge, United Kingdom), TREM2 (1:300, AF1729, R&D System), Gal-3 (1:300, AF1197, R&D System), CGRP (1:1000, #14959, Cell Signaling), IB4 (1:1000, #13250, Thermo Fisher Scientific), Neu-N (1:1000, #12943, Cell Signaling), GFAP (1:100, DakoCytomation, Glostrup, Denmark), MAP2 (1:2000, ab183830, Abcam, Cambridge, United Kingdom), CD11c (1:500, #97585, Cell Signaling).<br><br>The following antibodies were used for flow cytometry or fluorescence-activated cell sorting:<br>CD45 BV785 (0.5 µg/ml, clone 30-F11, 103149, Biolegend), CD11b BV421 (0.5 µg/ml, clone M1/70, 101251, Biolegend), CX3CR1-AF488 (1 µg/ml, clone SA011F11, 149022, Biolegend), P2Y12-PE (1 µg/ml, clone S16007D, 848004, Biolegend), TLR4-PECy7 (1 µg/ml, clone SA15-21, 145408, Biolegend), MHCII-PerCp/Cy5.5 (1 µg/ml, clone M5/114, 107626, Biolegend), CD11c-BV650 (2 µg/ml, clone N418, 117339, Biolegend), MerTK-BV605 (1 µg/ml, clone 2B10C42, 151517, Biolegend), CCR2-BV711 (0.5 µg/ml ;Clone 475301, 747848, BD Bioscience), Ly6C-APC (1 µg/ml, clone HK1.4, 17-5932-82, Thermo Fisher Scientific).
Validation	All antibodies were commercial in origin and validated by the company. Information regarding validation processes can be easily accessed on the company's web site using the product numbers listed above.

Animals and other research organisms

Policy information about studies involving animals; ARRIVE guidelines recommended for reporting animal research, and Sex and Gender in Research

Laboratory animals	All experiments were performed on 6-month-old male and female C57BL/6J wild type (WT) and heterozygous TASTPM (APPswe x PS1.M1466V) mice. The colonies were bred in house. WT mice were generated by backcrossing heterozygous TASTPM mice to produce mice carrying no human transgene. All animals were housed in the Biological Services Unit, King's College London; maintained at room temperature with a 12hr light/dark cycle, in groups of up to 5 per standard cage, with access to food and water ad libitum. TLR4 KO mice were kindly provided by Dr S. Akira, Osaka, Japan
Wild animals	The study did not involve wild animals.
Reporting on sex	Statistically well-powered behavioral experiments were conducted in both male and female mice with no significant differences observed. Following, for the molecular and transcriptomics experiments equal number of male and female mice were used.
Field-collected samples	The study did not involve samples collected from the field.
Ethics oversight	All procedures were performed under personal and project licenses and complied with the United Kingdom Animal Scientific Procedures Act 1986. Approval for studies was provided by King's Animal Welfare and Ethical Review Body, London, United Kingdom.

Note that full information on the approval of the study protocol must also be provided in the manuscript.

Flow Cytometry

Plots

Confirm that:

- [x] The axis labels state the marker and fluorochrome used (e.g. CD4-FITC).
- [x] The axis scales are clearly visible. Include numbers along axes only for bottom left plot of group (a 'group' is an analysis of identical markers).
- [x] All plots are contour plots with outliers or pseudocolor plots.
- [x] A numerical value for number of cells or percentage (with statistics) is provided.

Methodology

Sample preparation	To isolate cells for bulk RNA-seq, mice were euthanized with pentobarbital and were subsequently transcardially perfused

	using ice-cold PBS 1x and the brains and spinal cords were quickly dissected and placed in PBS on ice. Microglia/ myeloid cell extraction was carried out following a published protocol. The whole procedure was done on ice with cold buffers containing RNase inhibitors (N2615, Promega). Briefly, the tissue was dounce homogenized in ice-cold PBS with loose and tight pestles. The cell suspension was then transferred through a pre-wet 70µm cell strainer (BD Falcon). Myelin removal was performed by adding 100ul Myelin Removal Beads II (Miltenyi Biotec) per dorsal spinal cord and loaded in pre-wet LS columns (Miltenyi Biotec) on a MACS magnet stand separator. Cells in the flow through were collected and washed for standard FACS staining.<br><br>To sort microglia using FACS, cells were incubated with anti-mouse CD16/CD32 antibodies (BD Biosciences) for 15 min on ice. Before antibody staining, samples were incubated with the Zombie NIR™ Fixable Viability Kit (Biolegend) that identifies dead cells in the suspension that are later discarded during FACS. Then cells were incubated with covalently conjugated to fluorochromes for 45 min on ice on shaker. Finally, cells were resuspended in 300µl FACS buffer and 30ul of Precision Count Beads™ (Biolegend) were added to quantify the number of cells expressing markers of interest. Samples were run using BD FACSAria™ II (BD Bioscience) and analysed using FlowJo software (v10.1; Tree Start Inc.). Cells were sorted directly into a 1.5ml Eppendorf tube containing 350ul RLT buffer before subsequent RNA extraction.
Instrument	BD FACSAria™ II
Software	FlowJo software (v10.1; Tree Start Inc.)
Cell population abundance	Abundance is reported in Figure 3a, with microglia purity of more than 99%.
Gating strategy	Gating strategy is shown in Supplementary figure 3. Briefly, cells were defined as microglia when they were CD45low, CD11b +, CCR2neg, Ly6Cneg, CX3CR1+.

☒ Tick this box to confirm that a figure exemplifying the gating strategy is provided in the Supplementary Information.
